# The Arabidopsis RNA Binding Protein with K Homology Motifs, SHINY1, Interacts with the C-terminal Domain Phosphatase-like 1 (CPL1) to Repress Stress-Inducible Gene Expression

**DOI:** 10.1371/journal.pgen.1003625

**Published:** 2013-07-11

**Authors:** Jiafu Jiang, Bangshing Wang, Yun Shen, Hui Wang, Qing Feng, Huazhong Shi

**Affiliations:** Department of Chemistry and Biochemistry, Center for Chemical Biology, Texas Tech University, Lubbock, Texas, United States of America; University of California Riverside, United States of America

## Abstract

The phosphorylation state of the C-terminal domain (CTD) of the RNA polymerase II plays crucial roles in transcription and mRNA processing. Previous studies showed that the plant CTD phosphatase-like 1 (CPL1) dephosphorylates Ser-5-specific CTD and regulates abiotic stress response in Arabidopsis. Here, we report the identification of a K-homology domain-containing protein named SHINY1 (SHI1) that interacts with CPL1 to modulate gene expression. The *shi1* mutant was isolated from a forward genetic screening for mutants showing elevated expression of the luciferase reporter gene driven by a salt-inducible promoter. The *shi1* mutant is more sensitive to cold treatment during vegetative growth and insensitive to abscisic acid in seed germination, resembling the phenotypes of *shi4* that is allelic to the *cpl1* mutant. Both SHI1 and SHI4/CPL1 are nuclear-localized proteins. SHI1 interacts with SHI4/CPL1 *in vitro* and *in vivo*. Loss-of-function mutations in *shi1* and *shi4* resulted in similar changes in the expression of some stress-inducible genes. Moreover, both *shi1* and *shi4* mutants display higher mRNA capping efficiency and altered polyadenylation site selection for some of the stress-inducible genes, when compared with wild type. We propose that the SHI1-SHI4/CPL1 complex inhibits transcription by preventing mRNA capping and transition from transcription initiation to elongation.

## Author Summary

Plants, including important economic crops, frequently grow under unfavorable conditions that largely reduce their production potential. Plants respond to these stress conditions by adjusting physiological status resulting from changes in gene expression. Many genes that are repressed at normal growth conditions are activated in response to stresses. The presented work here attempted to answer the question as to how stress inducible genes are repressed at normal growth conditions. We established a genetic system to identify genes that are essential for such repression and found a protein complex that plays crucial roles in regulating stress inducible gene expression. Our work also revealed important molecular processes that are modulated by the identified protein complex. These findings will help our understanding about gene expression and regulation in general and the molecular mechanisms governing plant stress response in particular.

## Introduction

In eukaryotes, gene transcription includes several co-transcriptional processes such as mRNA 5′ capping, splicing and polyadenylation. These co-transcriptional processes are executed by protein enzymes and factors that are recruited to the carboxyl terminal domain (CTD) of the largest subunit of RNA polymerase II (Pol II) during gene transcription. The factors to be recruited to the CTD are determined by the phosphorylation patterns of the highly conserved tandem repeats (Y^1^S^2^P^3^T^4^S^5^P^6^S^7^) in the CTD that is regulated by site-specific CTD kinases and phosphatases [1]. Phosphorylation status of the Ser-2 and Ser-5 in the heptapeptide repeat of the CTD is thought to be important for the co-transcriptional processes and recycling of the RNA polymerase II. 5′capping of the nascent transcript occurs shortly after the transcription initiation and requires CTD phosphorylation at the Ser-5 by the general transcription factor TFIIH [1], [2], while phosphorylation of the Ser-2 residues by the positive transcription elongation factor b (P-TEFb) is believed to promote transcription elongation, splicing, and 3′end processing [3]. Recycling of Pol II requires dephosphorylation of the CTD and several CTD phosphatases are known to function in this process. In yeast, SCP1 [4], [5] and Ssu72 [6] are for the dephosphorylation of Ser-5, while FCP1 is for the dephosphorylation of Ser-2 [7]. However, in *Encephalitozoon cuniculi*, Fcp1 dephosphorylates both Ser-5 and Ser-2 [8].

The Fcp1 protein has a conserved N-terminal Fcp1 homology (FCPH) region with the DXDX (T/V) signature motif important for its catalytic activity. In Arabidopsis, the FRY2/CPL1 protein also possesses the FCPH domain at its N-terminus that is essential for its CTD phosphatase activity. FRY2/CPL1 dephosphorylates Ser-5 rather than Ser-2 in the CTD repeat polypeptides *in vitro* [9], [10]. In addition, the FRY2/CPL1 contains two dsRNA binding domains at its C-terminus that may be important for its association with RNA. FRY2/CPL1 was initially identified in genetic screenings in Arabidopsis for mutants showing altered expression of luciferase reporter gene driven by the stress-inducible promoter *Rd29A*. The *fry2* mutant was recovered from an EMS mutagenized population [11] and the *cpl1* mutant was identified from a T-DNA insertion mutagenized population [12]. Mutations in *FRY2/CPL1* resulted in significant increases in the expression of luciferase reporter gene and other stress-responsive genes, indicating that FRY2/CPL1 is a repressor for stress inducible genes. *fry2/cpl1* mutant plants do not show apparent growth and development phenotypes under normal growth conditions except that the mutants flower later than wild type [11], [12]. However, *fry2* mutants display phenotypes in response to salt, ABA, freezing treatments, low iron availability and cadmium toxicity [11], [13]. It was proposed that FRY2/CPL1 functions as a negative regulator of gene expression by inhibiting the formation of elongation complex or mRNA capping via dephosphorylation of the Ser-5-PO4 of the CTD within the initiation complex or the early elongation complex [9]. Moreover, a recent study using fast-forward genetics has identified the CPL1 as an important player in miRNA biogenesis [14]. In this study, the CPL1 was shown to interact with and dephosphorylate HYL1 within the miRNA microprocessor complex, which is required for accurate miRNA processing and strand selection. Studies so far on FRY2/CPL1 have suggested that this CTD phosphatase is a multi-functional protein involving in different cellular and biochemical processes.

During transcription and post-transcriptional processes, mRNAs are always associated with RNA-binding proteins (RBPs) [15]–[17]. Generally, RBPs have one or more RNA-binding domains of the RNA recognition motif (RRM), the K-homology (KH) domain, or the combination of these two most widely present RNA-binding domains [15], [18], [19]. The KH domain was named due to its first discovery from the human protein heterogeneous nuclear ribonucleoprotein K (hnRNP K) [20]. KH domains were then found in many other proteins functioning in diverse processes including transcription, mRNA stability, translational silencing and mRNA localization [21]. KH domain proteins have been implicated in human diseases. For example, fragile X mental retardation syndrome is caused by lack of functional fragile X mental retardation protein (FMRP) containing two KH domains. A single mutation (Ile304 to Asn) in the KH2 of FMRP is responsible for this syndrome in a particularly pernicious case [22], [23]. The KH splicing regulator protein/fuse binding protein 2 (KSRP/FBP2) contains four KH domains and plays important roles in ARE mediated mRNA decay [24]. In Arabidopsis, 26 KH domain-containing proteins were found through sequence analysis [18]. Up to now, only three of the 26 predicted KH domain-containing proteins have reported functions. The first KH domain-containing protein with a reported function is HEN4 (HUA ENHANCER 4) [25]. HEN4 contains five or four KH domains depending upon exclusion or inclusion of the last intron in the transcripts due to alternative splicing. HEN4, together with HUA1 and HUA2, promote *AGAMOUS* pre-mRNA processing and play a critical role in floral morphogenesis [25]. Another KH domain containing protein in Arabidopsis is FLK (Flowering Locus KH Domain) with three KH domains and acts as a repressor of *FLC* gene to regulate flowering time [26]. The three KH domain containing protein PEP in Arabidopsis functions in controlling vegetative growth and pistil development by interacting with elements of the *CLAVATA* signaling pathway [27].

In this paper, we present a study of a previously uncharacterized KH domain containing protein named SHINY1 (SHI1) in Arabidopsis. The *shi1* mutant was isolated from a forward genetic screening for mutants showing elevated expression of the luciferase reporter gene driven by a salt inducible promoter. We show here that SHI1 interacts with the CTD phosphatase FRY2/CPL1 to modulate co-transcriptional processes such as mRNA capping and polyadenylation. The SHI1-FRY2/CPL1 complex, together with other unidentified protein components, functions to repress stress-inducible gene expression.

## Results

### Identification of the *SHI1* Locus

In an attempt to identifying regulators of stress-inducible genes, we established a forward genetic screening for mutants showing elevated expression of the luciferase reporter gene driven by a salt inducible promoter from the sulfotransferase gene *AtSOT12* (*At2g03760*). The *AtSOT12* gene expression is highly induced by salt and osmotic stress according to our previous finding [28] and a microarray analysis [29]. To test whether the *AtSOT12* promoter is a salt-inducible promoter, a chimeric gene consisting of the firefly luciferase gene driven by the *AtSOT12* promoter was transformed into Arabidopsis ecotype Columbia-0. A homozygous transgenic line showing normal morphology, growth and development was selected for further study. Both Northern hybridization and luciferase imaging revealed that the luciferase expression is highly induced by salt stress (Figure S1) in the homozygous line, indicating that the *AtSOT12* promoter is indeed a salt inducible promoter. Seeds of the homozygous transgenic line (referred to as wild type) were mutagenized with EMS and the M2 seeds from 20 pools were subjected to mutant screening using the luciferase imaging system. A number of mutants showing higher expression of luciferase after salt stress treatment were identified. These mutants were named *shiny* (*shi* in short) mutants because of their bright luminescence imaging. One *shiny* mutant, designated *shi1*, is studied in the present paper.

**Figure 1:**
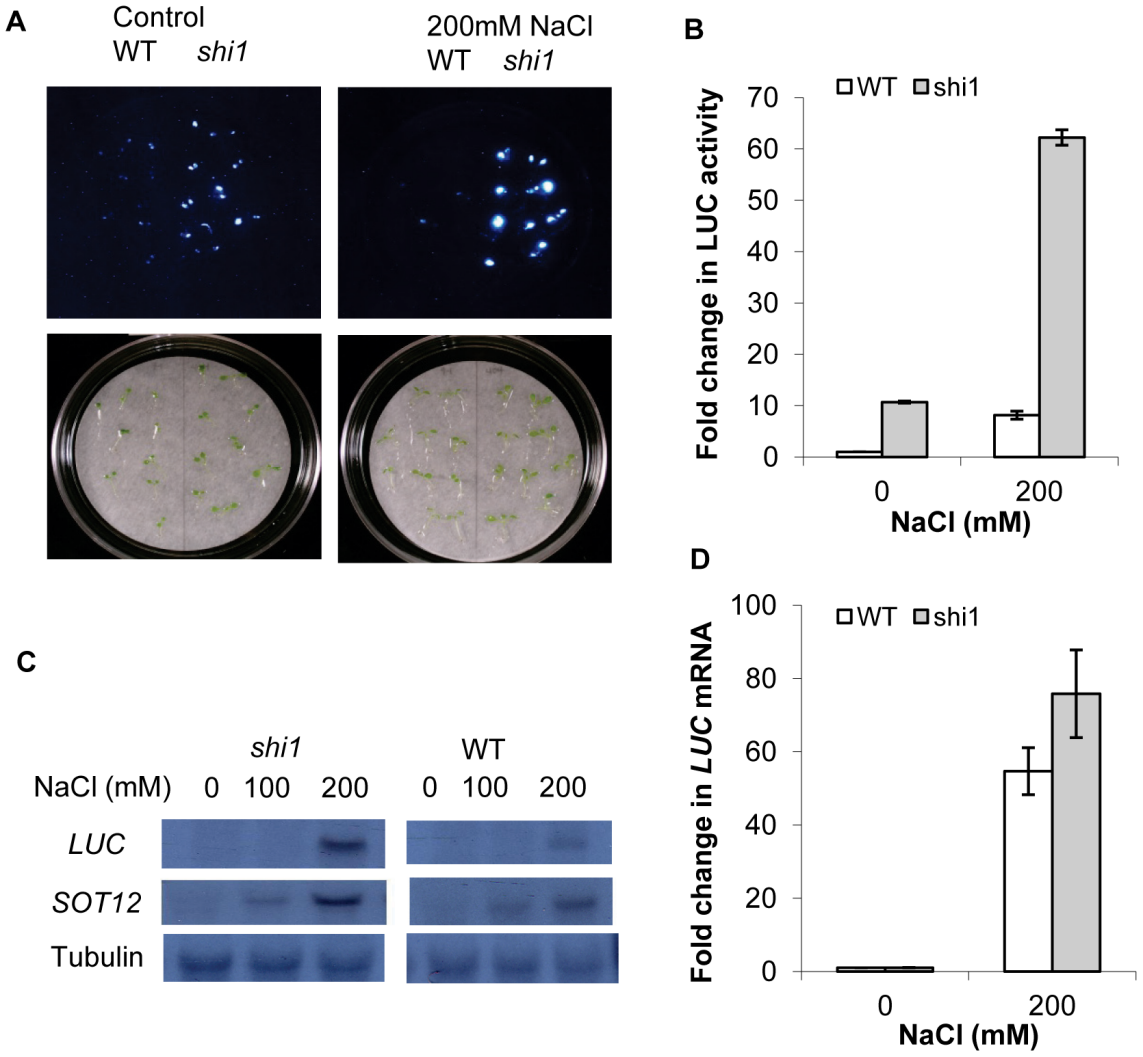
Characterization of the *shi1* mutant. (A) Luciferase imaging of 7-day-old seedlings with or without 200 mM NaCl treatment. (B) Quantitative measurements of luciferase activity. Values are means ± SD (n = 10). (C) Northern blot showing luciferase and *AtSOT12* gene transcript levels. Tubulin is shown as a loading control. (D) Quantitative measurement of luciferase transcript levels by quantitative RT-PCR. Values are means ± SD (n = 3).

As shown in Figure 1A, the *shi1* mutant displayed an elevated luciferase expression comparing with the wild type under both normal condition and after salt treatment. Quantitative analysis of luminescence intensity further confirmed that luciferase activity in the *shi1* seedlings was much higher than that in the wild type with or without NaCl treatment (Figure 1B). To determine whether the increased luciferase activity in *shi1* mutant was due to increased luciferase transcript level, RNA blot analysis was carried out. Figure 1C shows that the luciferase gene transcript was not detectable in both wild-type and *shi1* mutant plants without stress treatment, whereas *shi1* mutant exhibited substantially higher luciferase transcript level than the wild type after 200 mM NaCl treatment for 5 hours. Quantification of the transcript levels indicated that the fold change of luciferase transcript is much lower than the fold change of luciferase activity (Figures 1B and 1D), which suggests that the increased transcription level of luciferase gene only partly contributes to the increase luciferase activity in *shi1* mutant.

### The *shi1* Mutation Alters ABA and Cold Responsiveness

**Figure 2:**
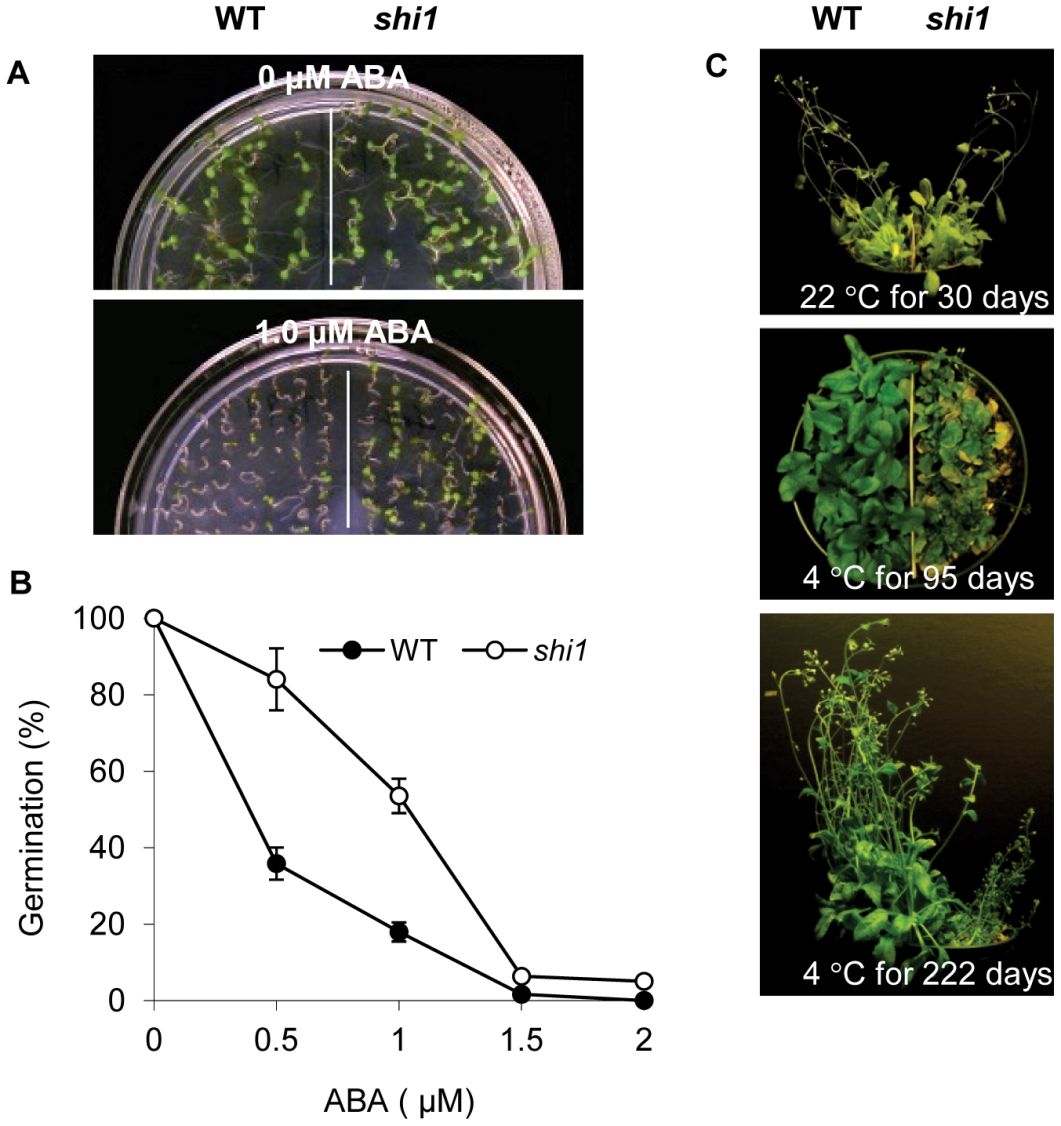
Stress response phenotype of the *shi1* mutant. (A) Seed germination with or without 1.0 µM ABA. (B) Seed germination at different concentrations of ABA. Values are means ± SD (n = 3). (C) Plant growth at room temperature and low temperature for indicated days.

After extensive phenotyping of the *shi1* mutant in response to different abiotic stresses and plant hormones, the *shi1* mutant was found to be more resistant to ABA in seed germination and more sensitive to low temperature during vegetative growth (Figure 2). The germination rate of *shi1* seeds at 7 d was reduced to 84.0% relative to 35.8% of the wild-type seeds in the presence of 0.5 µM ABA. When the concentration of ABA increased to 1.0 µM, the germination rate of *shi1* decreased further to 53.5% comparing with 17.9% of the wild-type seeds (Figure 2B). Under normal growth conditions, *shi1* plants essentially resembled the wild type plants in growth and development (Figure 2C). However, when growing under cold condition at ∼4°C with 16 h light/8 h dark, the *shi1* mutant plants showed yellow leaves and smaller rosette comparing with the wild-type plants (Figure 2C). The flowering *shi1* plants exhibited apparent smaller size comparing with the flowering wild type plants when growing under such cold condition (Figure 2C). We also tested other stress treatments, such as ABA, NaCl, LiCl, mannitol and UV-light on the root growth and the *shi1* mutant showed similar response to these stress conditions with wild type.

### Expression of Stress-Responsive Genes in *shi1* Mutant

**Figure 3:**
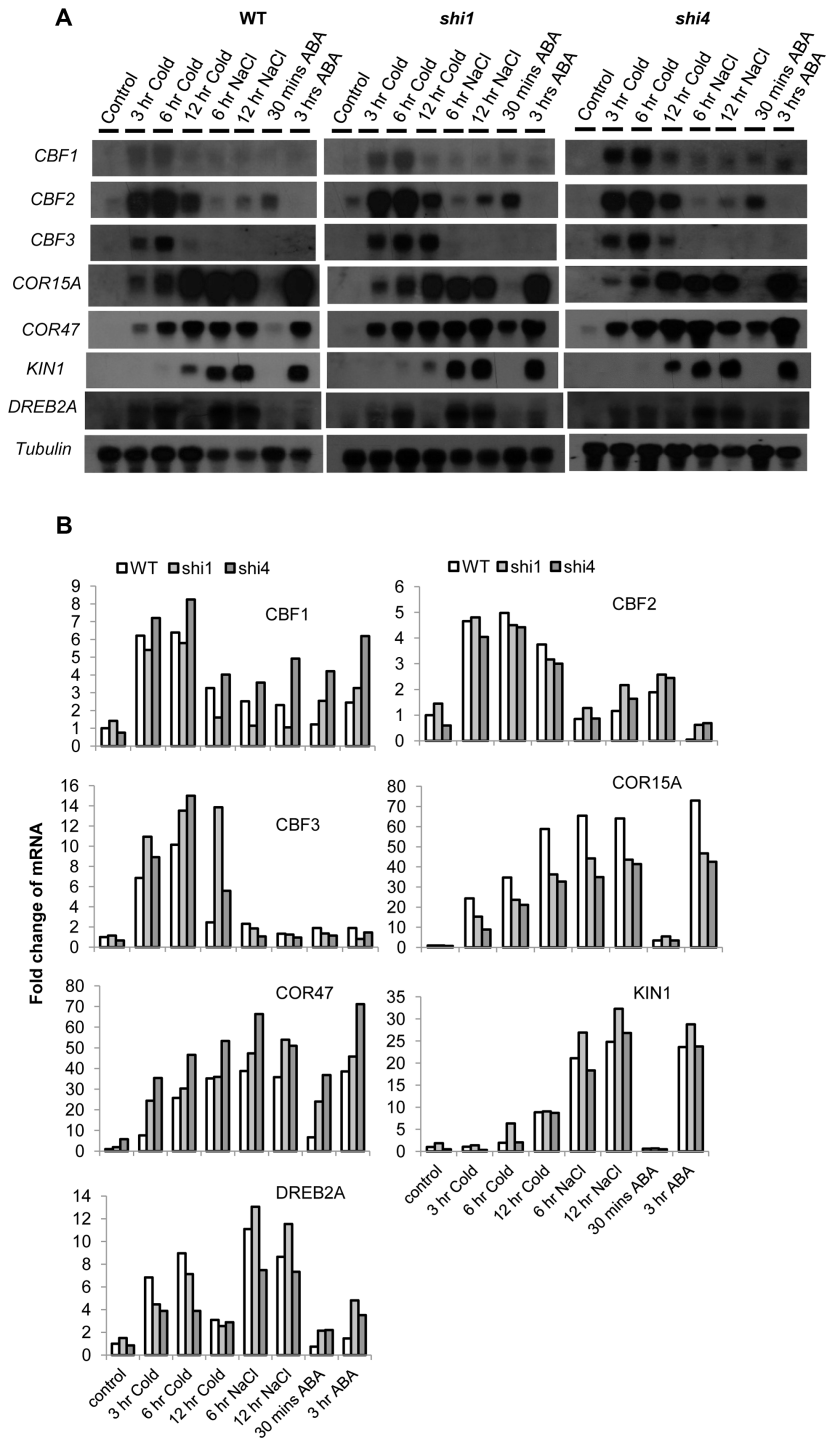
Detection of stress-inducible gene expression in wild type, *shi1* and *shi4* mutants. (A) Northern blot showing the transcript levels of stress-inducible genes in wild type, *shi1* and *shi4* mutants. Tubulin is shown as a loading control. (B) Quantitative measurements of the relative signal strengths compared with the signal strength in the wild type control. The signal strengths were measured by Imaging J. Each signal strength was normalized with the signal strength of the corresponding tubulin.

To determine the effects of the *shi1* mutation on stress-responsive gene expression, we selected a set of genes, including *CBFs*, *COR* genes and *DREB2A* that have been used as marker genes for cold, ABA and salt response [11]. The transcript levels of these stress-responsive genes were analyzed by using RNA blot. As shown in Figure 3, *CBF3* transcript levels were higher in the *shi1* mutant than in wild type plants after cold treatments. At the time point of 12 hours of cold treatment, the *shi1* mutant still showed strong induction of *CBF3* gene expression, while wild type did not. *CBF2* expression levels were similar between the *shi1* and wild type after cold treatments, while *shi1* mutant exhibited slightly higher *CBF2* transcript levels than the wild type plants when treated by NaCl or ABA. The *shi1* mutation had opposite effect on the expression of two *COR* (cold-responsive) genes, *COR15A* and *COR47*. The expression of *COR15A* was substantially lower in the *shi1* mutant than in the wild type, while the expression of *COR47* was higher in the *shi1* mutant than in the wild type under some tested stress conditions (Figure 3). The major differences in induced gene expression of *CBF3*, *COR15A* and *COR47* were further verified by using quantitative RT-PCR (Figure S2). These data indicate that SHI1 is involved in the regulation of abiotic stress responsive genes.

### Map-Based Cloning of the *SHI1* Locus

**Figure 4:**
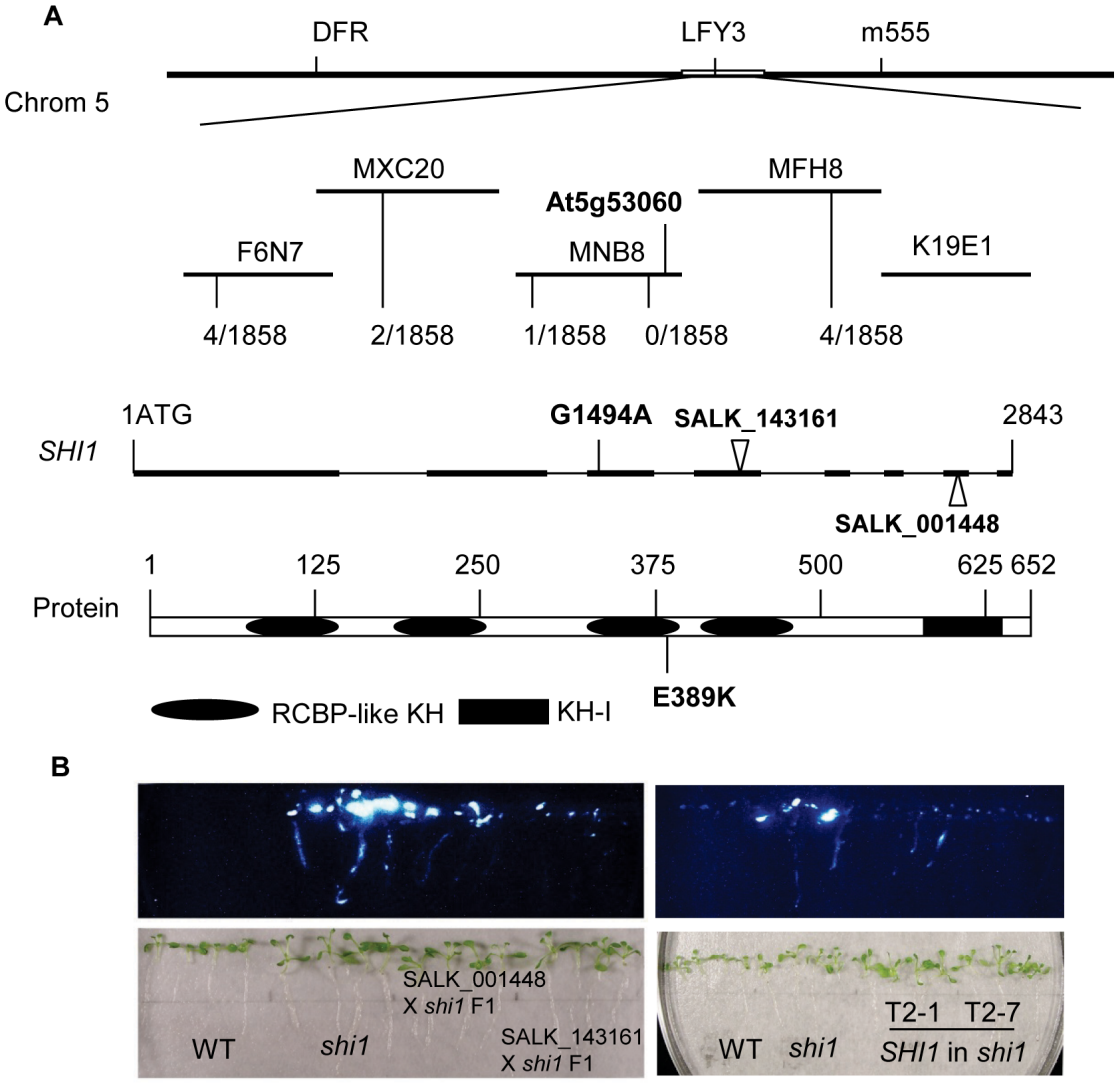
Map-based cloning of the *SHI1* locus. (A) Identification of the *shi1* mutation. Recombination events from 1858 chromosomes analyzed for each marker are shown. The locations of the *shi1* mutation of G1494A and two T-DNA insertions are shown. (B) Complementation tests for the *shi1* mutation. Left panel, genetic complementation between the *shi1* mutation and the T-DNA knockouts; Right panel, molecular complementation of the *shi1* mutation by the wild type *SHI1* gene.

Genetic analysis verified that the *shi1* mutation is a single nuclear recessive mutation. To determine the molecular identity of the *SHI1* gene, map-based cloning was employed using F2 seeds of the *shi1* mutant crossed with *Landsberg erecta* wild type as a mapping population. Mutant seedlings from the F2 mapping population were selected and the *shi1* mutation was mapped with simple sequence length polymorphism (SSLP) markers. The mutation was first mapped to the Chromosome 5 and then further narrowed down to between the BAC clones MNB8 and MFH8 (Figure 4A). After sequencing several candidate genes within this region, a nucleotide change of G1494A was found in the *At5g53060* gene in the *shi1* mutant. This nucleotide change resulted in an amino acid substitution of E369K in the third KH domain of this KH domain-containing protein (Figure 4A). To confirm whether the mutation in *At5g53060* is responsible for the phenotype of *shi1* mutant, the *shi1* mutant was crossed with two T-DNA lines SALK_143161 and SALK_001448 with T-DNA insertions in the *SHI1* gene. The F1 seedlings resulted from these genetic crosses displayed elevated luciferase expression comparing with the wild type (Figure 4B). For molecular complementation assay, a genomic fragment containing the *SHI1* open reading frame along with 1910 bp promoter sequence upstream of the translation start codon (corresponding to position 21513445, Chromosome5) and 262 bp of sequence downstream of the translation stop codon (corresponding to position 21518401, Chromosome5) was amplified from the BAC clone MINB8 and introduced into the *shi1* mutant by using the *Agrobacterium*-mediated floral dip transformation method [30]. The wild type SHI1 gene recovered the luciferase expression level of the *shi1* mutant to the level in the wild type (Figure 4B), which further supports that the *shi1* mutation in *At5g53060* gene is indeed responsible for the *shi1* mutant phenotype.

### Expression Pattern of the *SHI1* Gene

**Figure 5:**
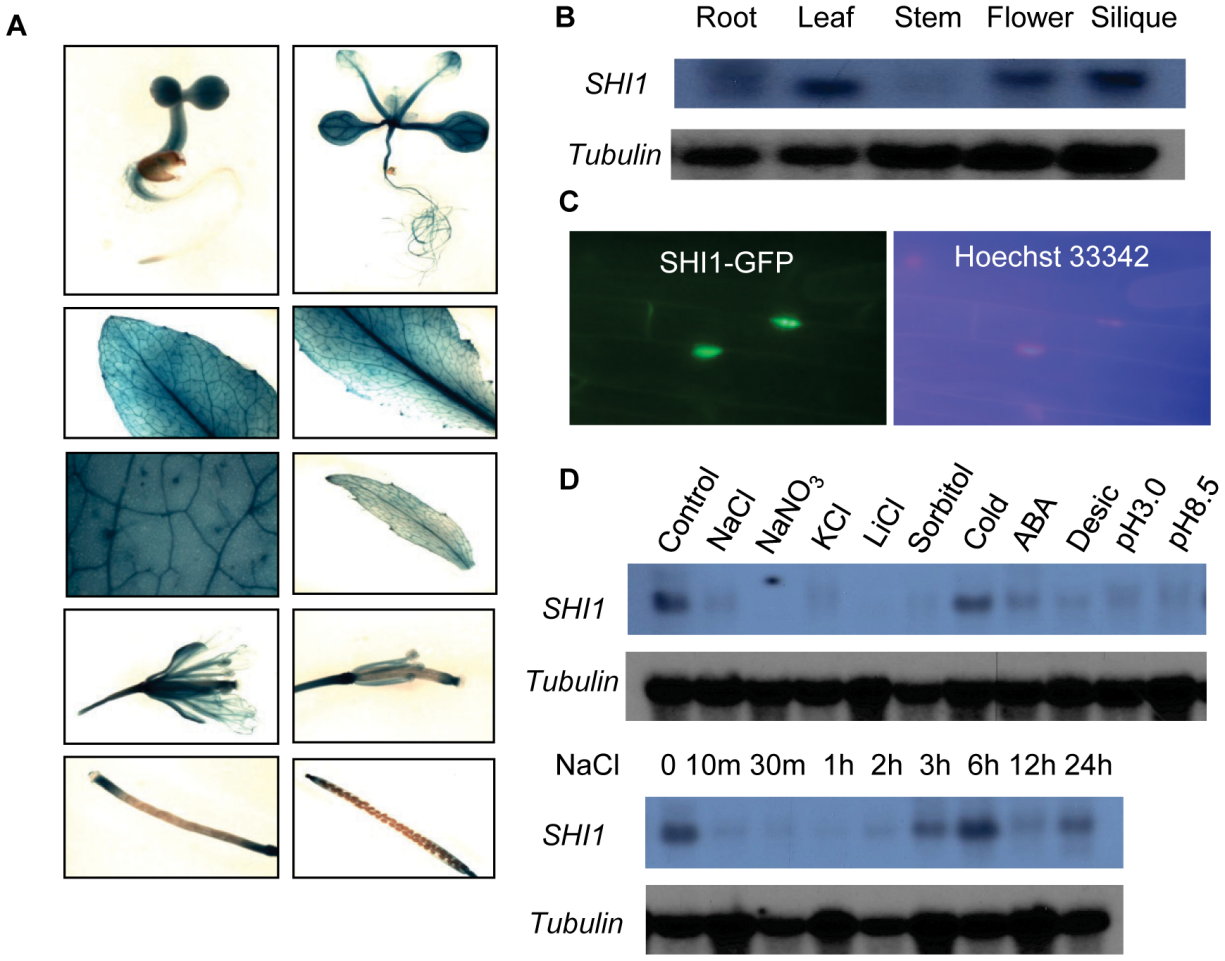
Gene expression and subcellular localization of the SHI1. (A) Promoter-GUS analysis showing GUS expression at different developmental stages and different organs. (B) Northern blot showing the transcript levels of *SHI1* gene in different organs. (C) The subcellular localization of the SHI-GFP fusion protein. The left panel shows the nuclear localization of the fusion protein and the right panel shows fluorescent staining of the nucleus by Hoechst 33342. (D) Northern blot showing the *SHI1* gene expression in response to different abiotic stress treatments. Control, control without treatment; NaCl, 100 mM NaCl for 12 h; NaNO_3_, 100 mM NaNO_3_ for 12 h; KCl, 100 mM KCl for 12 h; LiCl, 100 mM LiCl for 12 h; Sorbitol, 200 mM sorbitol for 12 h; Cold, 4°C for 24 h; ABA, 100 µM ABA for 3 h; Desicc, desiccation for 15 min; pH 3.0, pH 3.0 for 12 h; pH 8.5, pH 8.5 for 12 h. Tubulin is shown as a loading control.

The expression of the *SHI1* gene in plant was analyzed by using promoter-GUS assay and RNA blot analysis. *SHI1* is expressed in roots, leaves, flowers, and siliques, but its expression is low in stems (Figures 5A and 5B). SHI1 is a nuclear-located protein, which was revealed by examining the SHI1-GFP fusion protein expressed in a transgenic Arabidopsis plant (Figure 5C). The expression of *SHI1* gene was sharply reduced by treatments with high concentrations of salt such as NaCl, NaNO_3_, KCl and LiCl, hyperosmotic stress treatment with sorbitol, and treatments with ABA, low or high pH (Figure 5D). However, cold treatment did not significantly change the *SHI1* expression level (Figure 5D). Interestingly, the effect of NaCl treatment on the *SHI1* expression appears to have a clock rhythm. *SHI1* gene expression was quickly and sharply reduced by NaCl treatment for 10 min and this inhibition lasted up to 2 hours of NaCl treatment. The *SHI1* expression level gradually recovered to the control level after salt treatment from 3 to 6 hours. After 12 hours NaCl treatment, the SHI1 transcript was again reduced to very low level and then recovered substantially after 24 hours of NaCl treatment (Figure 5D).

### SHI1 Interacts with FRY2/CPL1

**Figure 6:**
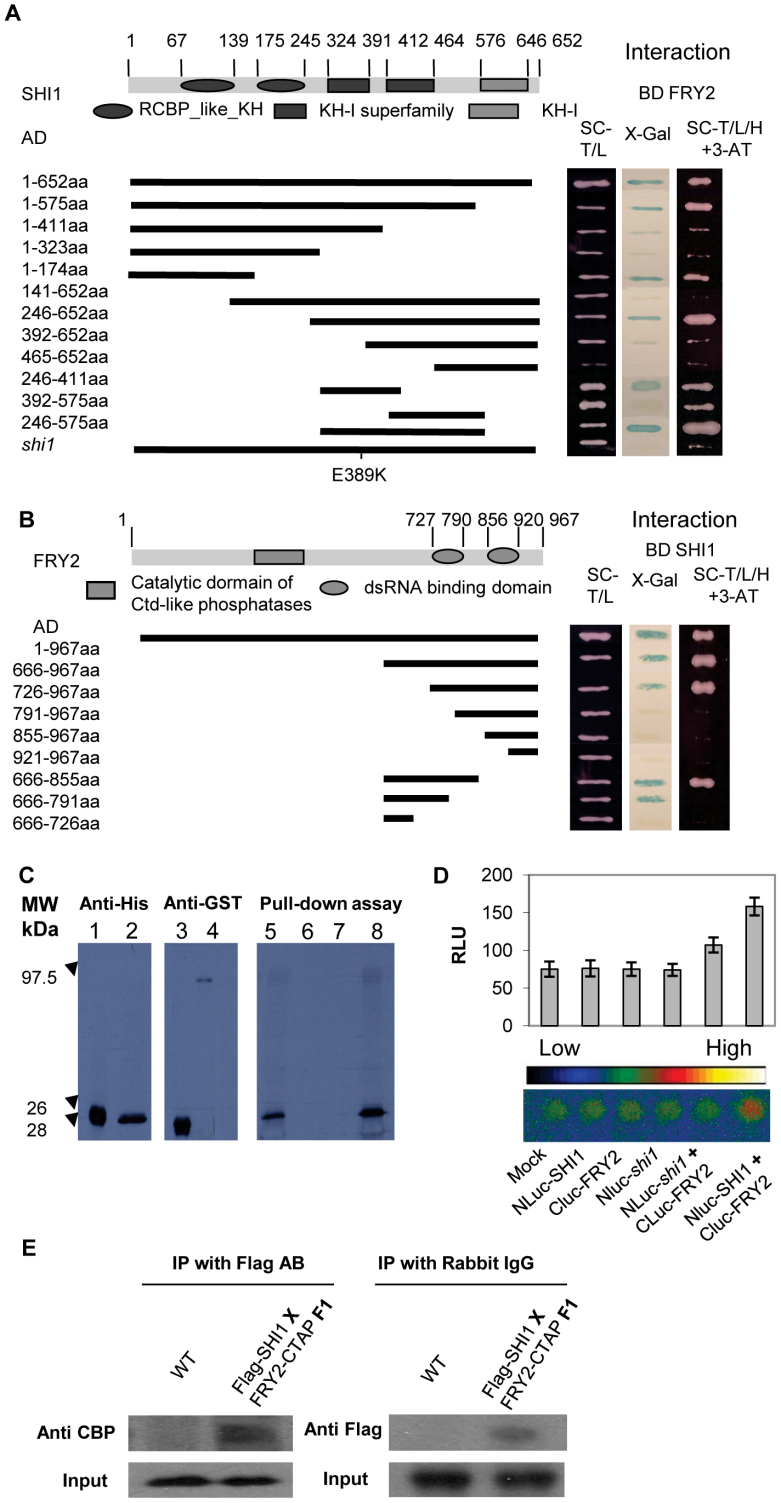
SHI1 and FRY2/CPL1 protein interaction analysis. (A) Yeast two-hybrid assays for detection of the domains in SHI1 required for its interaction with FRY2. Different deleted forms of SHI1 were fused with the activation domain (AD) and tested for their interactions with the full length FRY2 fused with the DNA binding domain (BD). A full length SHI1 with the *shi1* mutation was also included. The right panel shows yeast growth on the medium minus Trp, Leu and His with 3-amino-1,2,4-triazole (3-AT) and X-gal assays indicating the activation of the HIS and LacZ reporter genes due to protein-protein interaction. (B) Yeast two-hybrid assays for detection of the domain in FRY2 required for its interaction with SHI1. The deletion forms of FRY2 were fused with the AD and the full length SHI1 was fused with the BD. (C) Pull-down assay. Left panel, Western blot detecting *E. coli* expressed and *in vitro* translated HIS-tagged cFRY2 (666–855 aa) protein. 1, HIS-tagged cFRY2 expressed in *E. coli*; 2, *in vitro* translated HIS-cFRY2. Middle panel, Western blot detecting GST and GST-SHI1 fusion protein expressed in *E. coli*. 3, GST; 4, GST-SHI1 fusion protein. Right panel, Autoradiography showing pull-down assay. 5, *in vitro* translated and ^35^S-methionine labeled HIS-cFRY2 shown as input; 6, pull-down with resin only as a control; 7, pull-down with GST as a control; 8, pull-down with GST-SHI1 fusion protein. (D) Split-luciferase assay. Upper panel, quantitative analysis of relative luminescence unit (RLU). Values are means ± SE (n = 3). Lower panel, luciferase imaging. The *shi1* mutation was also included for protein interaction assay. (E) Co-immunoprecipitation assay.

To better understand the molecular function of SHI1 in Arabidopsis, a yeast two-hybrid screen for SHI1-interacting proteins was performed. 18 independent clones representing *FRY2/CPL1* cDNA showing interaction with SHI1 bait protein were identified from the prey cDNA library (TAIR Cat. No. CD4-30). Among the 18 clones, three different sizes of cDNAs were obtained: the longest cDNA corresponding to 476–967 aa sequence, the medium sized cDNA corresponding to 533–967 aa sequence, and the shortest cDNA corresponding to 666–967 aa sequence of the C-terminus of the FRY2/CPL1 protein. The full length protein of FRY2/CPL1 was also confirmed to interact with SHI1 in the yeast two-hybrid system (Figure 6B).

Protein deletion analysis was used to pinpoint the regions responsible for SHI-FRY2/CPL1 interaction. As shown in Figure 6A, the first KH domain and the third KH domain in the SHI1 protein could interact with FRY2/CPL1. The *shi1* mutation of Glu389 to Lysine change in the third KH domain disrupted the interaction of SHI1 with FRY2/CPL1, suggesting that the formation of SHI1-FRY2/CPL1 protein complex is essential for SHI function and loss-of-function of *shi1* mutation is probably due to disruption of such a complex formation. Deletion analysis also revealed that the first dsRNA binding motif is required for the interaction of FRY2/CPL1 with SHI1 (Figure 6B).

Direct physical interaction between SHI1 and FRY2/CPL1 was determined with protein pull-down assay. The amino acid sequence including the first dsRNA binding domain (666–855 aa) of the FRY2/CPL1 was fused with 6XHIS tag (HIS-cFRY2) and synthesized by using an *in vitro* translation system. GST-SHI1 tagged protein was expressed and purified from *E. coli* for *in vitro* pull-down assay. As shown in Figure 6C, the ^35^S-methonine radioactive labeled HIS-cFRY2 was pulled down together with GST-SHI1, which confirms a physical interaction between SHI1 and FRY2/CPL1. Interaction of SHI1 with FRY2/CPL1 in plant cells was further established by using a split luciferase complementation assay [31] and split YFP complementation assay. The SHI1 protein was fused with the N-terminal portion of the luciferase and the FRY2/CPL1 protein was fused with the C-terminal part of the luciferase and co-expressed in Arabidopsis protoplasts. Luciferase activity measurements indicated that co-expression of these two fusion proteins generated significantly higher luciferase activity than all controls tested (Figure 6D), which suggests that SHI1 can interact with FRY2/CPL1 in plant cells. Split-YFP assay also confirmed the interaction of SHI1 with FRY1 in plant protoplasts (Figure S3). Furthermore, co-immunoprecipitation assay was carried out to determine *in planta* interaction of these two proteins. Transgenic plants expressing FLAG-SHI1 and FRY2-c-TAP tagged proteins were created and homozygous transgenic lines were generated. The F1 plants resulting from the cross between FLAG-SHI and FRY2-c-TAP plants were used for protein isolation and co-immunoprecipitation. Figure 6E shows that SHI1 and FRY2/CPL1 could mutually co-precipitate in plants, which strongly suggests that these two proteins can form a protein complex in plant for gene regulation.

### Identification of *shi4* as an Allele of the *fry2/cpl1*

**Figure 7:**
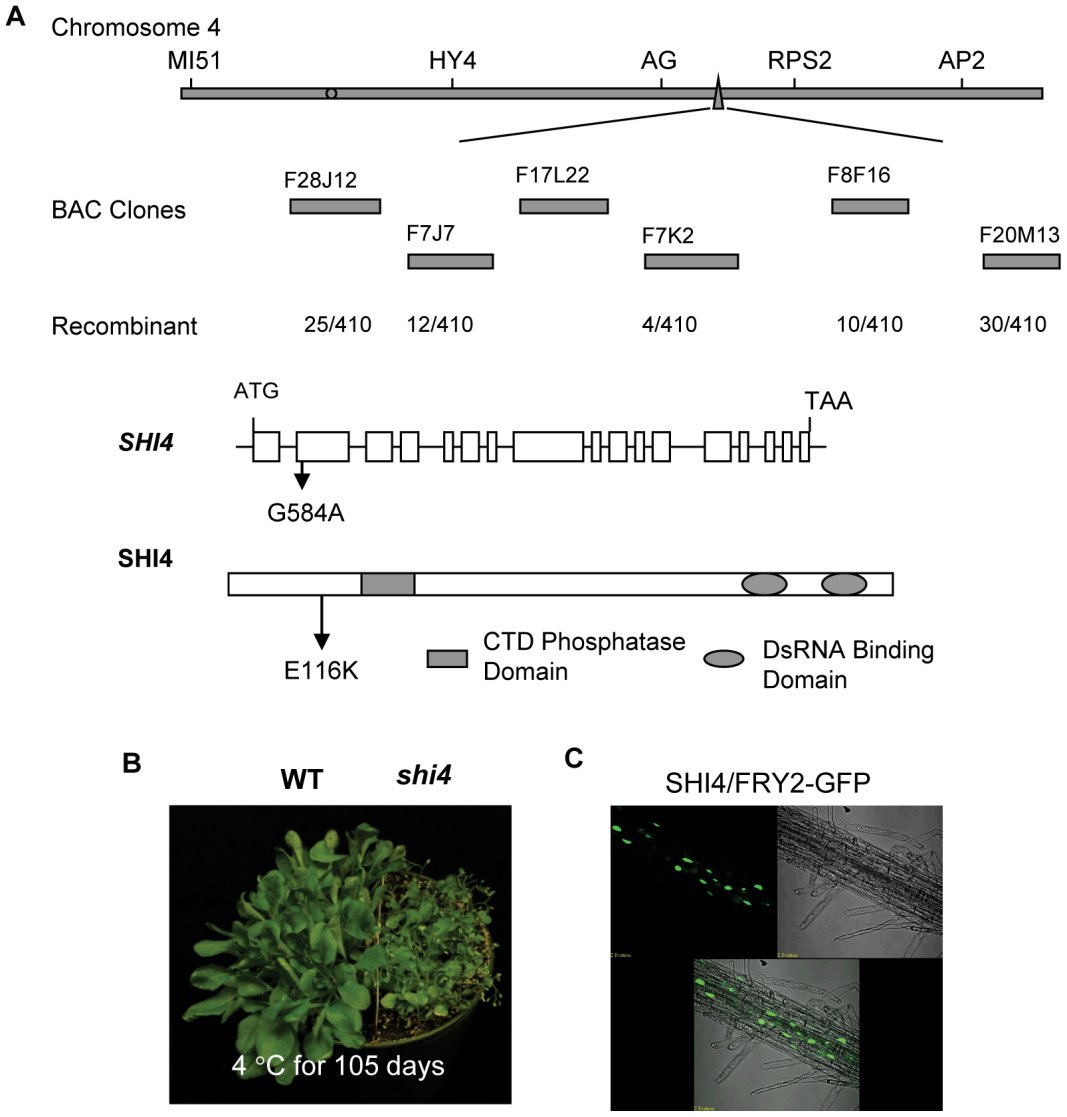
Map-based cloning and characterization of the *SHI4* locus. (A) Identification of the *shi4* mutation. (B) Hypersensitive phenotype of the *shi4* mutant to low temperature. (C) Subcellular localization of SHI4-GFP fusion protein. Showing is GFP fluorescence and merged image of GFP fluorescence in the root.

The *shi4* mutation was first located in the chromosome 4 and then narrowed down to the region between the BAC clones F7K2 and F7J7. In this region, the *FRY2*/*CPL1* gene (*At4g21670*) was previously identified as an important regulator of stress-responsive genes [11], [12], [32]. Sequencing of the *FRY2*/*CPL1* gene in the *shi4* mutant determined an allelic mutation of a G to A transition in the second exon resulting in an E116K substitution in the FRY2/CPL1 protein (Figure 7A). This recessive loss-of-function mutation in FRY2/CPL1 was also identified in a forward genetic screening for mutations altering wounding-induced gene expression [32].

The *shi4* mutant displayed elevated luciferase expression resembling the *shi1* and the previously reported *fry2*/*cpl1* mutants [11], [12]. Besides, the *shi4* mutant also exhibited cold sensitive phenotype similar to that of the *shi1* mutant (Figure 7B). Analysis of SHI4-GFP fusion protein revealed a nuclear localization of SHI4 (Figure 7C). Study of the expression of stress inducible genes in *shi4* indicated that both *shi4* and *shi1* mutations affected the expression of those cold, osmotic and ABA inducible genes in a similar way (Figure 3). Taken together, these results further support that SHI1 and SHI4/FRY2/CPL1 form a functional complex in Arabidopsis to regulate the expression of stress responsive genes.

### SHI1 and SHI4/FRY2/CPL1 Involves in mRNA Capping and Polyadenylation Site Selection

**Figure 8:**
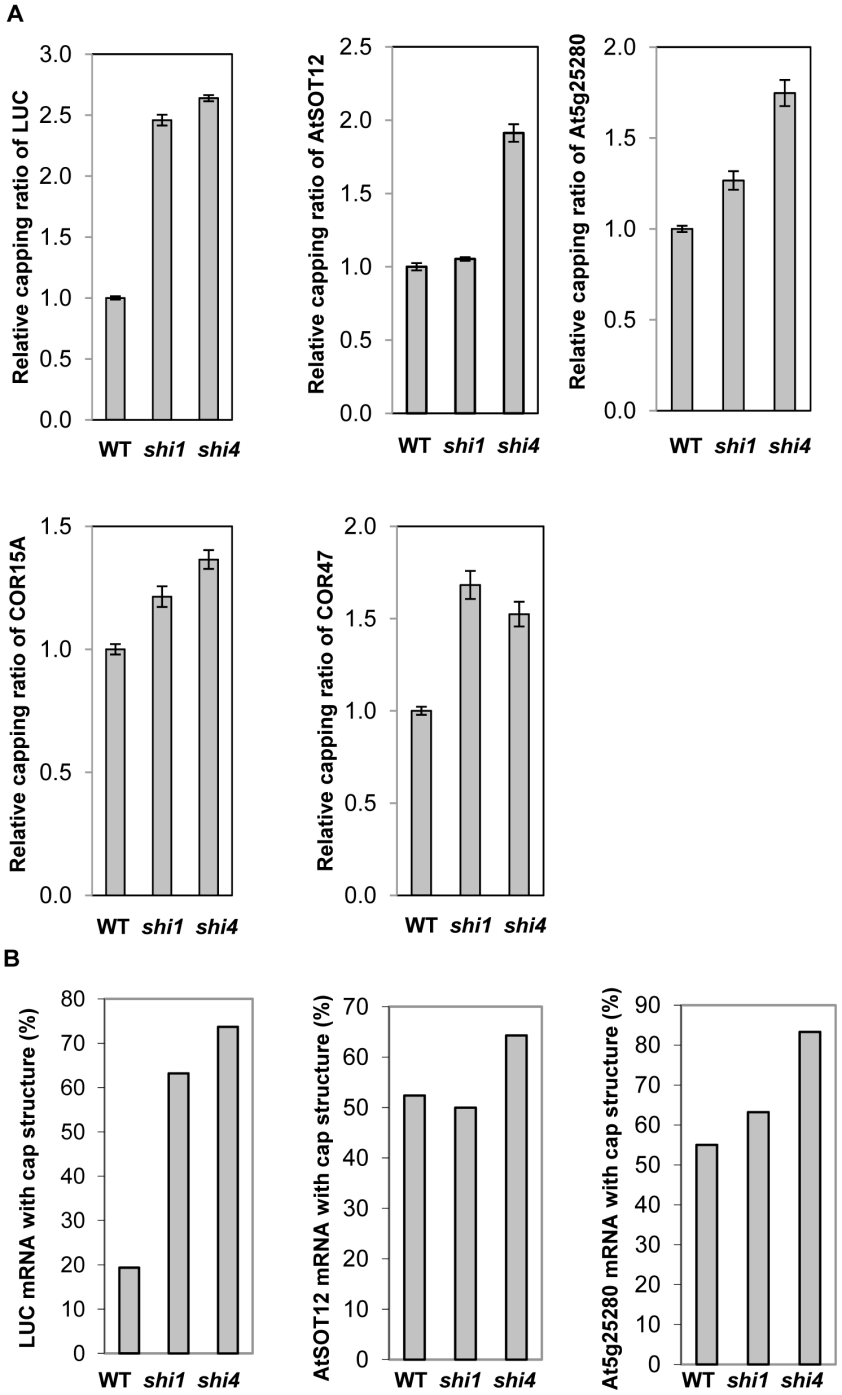
Detection of 5′ capping of the transcripts of *LUC*, *AtSOT12*, *At5g25280*, *COR15A*, and *COR47* in wild type, *shi1* and *shi4* mutants. (A) Relative capping ratios of the five selected genes transcripts determined by using the RLM-qRT-PCR method. Values are shown as mean ± SD (n = 3). (B) Percentages of the capped mRNA of three selected genes determined by using the 5′ RACE based method.

The FRY2/CPL1 protein has been shown to specifically dephosphorylate the Ser-5 at the CTD repeat of the RNA polymerase II [9], [10]. The Ser-5 phosphorylation is known to be required for recruiting the capping enzyme and stimulating mRNA capping [1], while dephosphorylation of the Ser-5 by CTD phosphatase has been shown to decrease mRNA capping [33]. To determine whether SHI1 and SHI4/FRY2/CPL1 are involved in mRNA capping, two methods were used to analyze the ratio of capped mRNA in total mRNA of individual genes. The first method was designed based on RNA Ligation Mediated Rapid Amplification of cDNA Ends (RLM-RACE) with modifications in which qRT-PCR was used instead of RACE. The total RNA was treated with Calf Intestine Alkaline Phosphatase to remove free 5′phosphate in the uncapped mRNA to prevent ligation of these mRNAs with the RNA adapter. The total RNA was then treated with Tobacco Acid Pyrophosphatase to remove the cap of the capped mRNA followed by a ligation of a RNA adapter with the treated RNA population. Only the de-capped mRNAs could be ligated with the RNA adapter. The RNA was reverse transcribed into cDNAs using random primers, and the ratio of capped transcripts in total transcripts of selected genes was determined by real-time PCR. This method was used to determine the relative mRNA capping ratio of five selected genes including the luciferase transgene, *AtSOT12*, *At5g25280* (a constitutively higher expressed gene in *shi1* and *shi4* that was found from our unpublished microarray data, Figure S4), *COR15A*, and *COR47*. As shown in Figure 8A, *shi4* mutant displayed substantially increased mRNA capping ratio of all five tested genes, while *shi1* mutant exhibited differential regulation of capping event in these five genes. For the *LUC* transgene and the endogenous stress-inducible gene *COR47*, *shi1* mutant showed increases in capping ratios comparable to that in *shi4* mutant. However, the *shi1* mutation did not alter the capping ratio of the endogenous *AtSOT12* mRNA and only caused marginal increases in capping ratios of *At5g25280* and *COR15A*. These results suggest that SHI4/FRY2/CPL1 is the major player in modulating mRNA capping and SHI1 protein is involved in capping of some mRNAs by partnering with SHI4.

The second method was a 5′RACE-based method to determine the 5′-m^7^G cap that can be reverse transcribed into a C in the first strand of the cDNA [34]. This method was first verified by detection of capped mRNA captured by the Arabidopsis cap-binding protein eIF4E (At4g18040) and uncapped mRNA incapable of binding by eIF4E. The Arabidopsis eIF4E was fused with GST and expressed and purified from *E. coli*. The binding specificity and kinetics of this fusion protein with capped mRNA were analyzed according to the previously published protocol [35]. The GST fused Arabidopsis eIF4E was found to specifically bind with capped mRNA and the binding constant is 0.18 nM (Figure S5). Purified GST-eIF4E proteins were incubated with Arabidopsis total RNA and capped mRNAs associated with the fusion protein were pulled down with the resin against the GST tag. 5′RACE results showed that 92% of the eIF4E-bound luciferase mRNAs have a 5′-cap that was reverse transcribed into the cDNA, while approximately 80% of the unbound luciferase mRNA did not show 5′ cap. This result supports that the 5′RACE based method is a reliable method for mRNA 5′ cap detection. Therefore, the 5′RACE-based method was used to detect the absolute ratio of capped transcripts in total transcripts of the selected genes. Figure 8B shows that the ratios of capped transcripts in total transcripts of luciferase, *AtSOT12*, and *At5g25280* genes in *shi4* mutant were significantly higher than that in the wild type, while *shi1* mutant showed significantly higher capping ratio of the luciferase mRNA, but exhibited no change in the endogenous *AtSOT12* mRNA capping and slight increase in *At5g25280* mRNA capping when compared with wild type. These results are consistent with the results showing in Figure 8A and strongly support that SHI1 and SHI4/FRY2/CPL1 act to negatively regulate mRNA capping.

**Figure 9:**
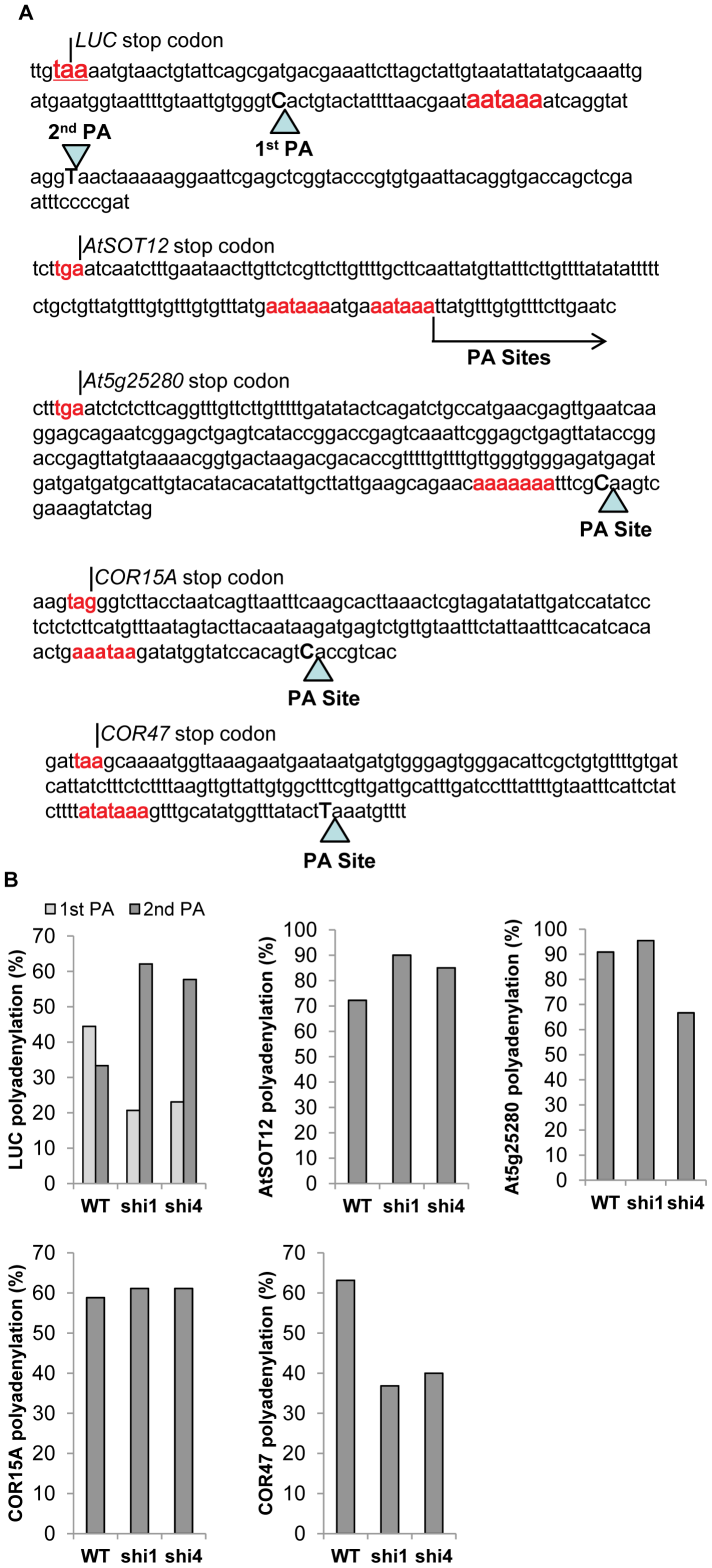
Detection of polyadenylation site selection of the transcripts of *LUC*, *AtSOT12*, *At5g25280*, *COR15A*, and *COR47* wild type, *shi1* and *shi4* mutants. (A) DNA sequences at the 3′ end of the five selected genes. Triangles indicate the major polyadenylation sites in the transcripts. Arrow shows the polyadenylation sites downstream of the indicated nucleotide in the *AtSOT12* transcripts. The stop codon and the putative polyadenylation signal sequence are highlighted in red. The last nucleotides at the polyadenylation sites are in uppercase and bolded. (B) Percentages of polyadenylation at the major polyadenylation sites in wild type, *shi1* and *shi4* mutants.

To determine whether SHI1 and SHI4/FRY2/CPL1 are involved in other co-transcriptional processes, a 3′RACE analysis was carried out to pinpoint the polyadenylation sites of the five selected genes transcripts. Major polyadenylation sites were found in four of the five genes, and the *AtSOT12* gene transcripts exhibited a dispersed pattern of polyadenylation sites mainly downstream of the putative polyadenylation signal sequences (Figure 9A). Two major polyadenylation sites were found in the luciferase mRNAs; one (designated 1^st^ PA) is located at the 21th position upstream of the canonical polyadenylation signal (cPA) sequence AAUAAA and the other (2^nd^ PA) at the position of the 13th nucleotide downstream of the cPA (Figure 9A). In wild type, polyadenylation at the 1^st^ and 2^nd^ PA sites were 44.4% and 33.3%, respectively. In *shi1* and *shi4* mutants, however, polyadenylation at the 1^st^ PA site was reduced to about 20%, while polyadenylation at the 2^nd^ PA site was increased to about 60% (Figure 9B). *AtSOT12* transcripts did not show a major polyadenylation site, but more than 70% of the *AtSOT12* transcripts in wild type displayed polyadenylation downstream of the cPAs. This ratio was significantly increased in the *shi1* and *shi4* mutants (Figure 9B). Both *shi1* and *shi4* mutations also strongly affected polyadenylation site selection in *COR47* transcripts. Polyadenylation at the major PA site of *COR47* in *shi1* and *shi4* mutants reduced remarkably when compared with that in wild type (Figure 9B). *shi4*, but not *shi1* mutant showed significant reduction in polyadenylation at the major PA site of *At5g25280*, and both mutants did not affect polyadenylation site selection of *COR15A* (Figure 9B). These results indicate that the *shi1* and *shi4* mutations have profound influence on PA site selection of the *LUC* transgene and also affect some endogenous genes. SHI1 is likely to be involved in some, but not all genes that are regulated by SHI4, as suggested by both capping and PA site selection analyses shown in Figure 8 and Figure 9.

## Discussion

Gene regulation is central for growth, development and adaptation to environmental changes in all living organisms. In plants, many genes are environmentally regulated, and proper response of these genes is critical for stress response and tolerance. Stress-inducible genes must be repressed or silenced at normal growth conditions but can be readily activated upon stress treatments. In the past decades, intensive studies have been focused on the activation mechanisms of inducible genes in response to stress conditions. Consequently, a number of *cis*-elements and transacting proteins required for gene induction have been identified [36]. For instance, the regulatory element DRE (dehydration responsive element) was identified from the drought, salt and cold inducible promoters and found to be recognized by the transcription factors DREB1/CBF and DREB2 [37]–[39]. However, little attention has been paid to the repression mechanisms of stress inducible genes at normal growth conditions. In this study, we utilized a luciferase-based mutant screening system to identify repressor proteins for stress inducible genes. We uncovered two repressor proteins, SHI1 and SHI4/FRY2/CPL1 that can form a complex and repress gene expression by modulating transcription and co-transcriptional processes. Interestingly, the SHI1 protein was also identified as a negative regulator of heat-inducible genes in a recent study using forward genetic screening for mutations affecting luciferase expression driven by the cold-inducible *CBF2* promoter [40]. Thus, SHI1 protein might be a general repressor for some of the stress-inducible genes. The cold and ABA responsive phenotypes of *shi1* and *shi4* protein could be attributed to mis-regulation of those cold and/or ABA-responsive genes as shown in Figure 3.

SHI1 protein belongs to the family of KH domain containing proteins comprising of 26 members in Arabidopsis. The nuclear localization of SHI1 (Figure 5C) indicated that SHI1 functions in a nuclear-based process. Moreover, we found that SHI1 can interact with the CTD phosphatase FRY2/CPL1, a previously characterized protein that dephosphorylates the Ser-5 at the CTD of the RNA polymerase II [9], [10]. Thus, it is conceivable that the SHI1 may modulate the CTD phosphorylation status through interacting with FRY2/CPL1, thereby regulating transcription and co-transcriptional processes. Functional interaction between SHI1 and FRY2/CPL1 was evidenced by the following findings. First, our genetic screening also recovered a *fry2*/*cpl1* allele named *shi4*, and *shi4* exhibited luciferase imaging and stress response phenotypes similar to the *shi1* mutant (Figure 7). Second, SHI1-FRY2/CPL1 interaction was verified by several protein interaction techniques including yeast two-hybrid assay, pull-down assay, split-luciferase assay and *in planta* Co-IP analysis (Figure 6). These analyses indicate that SHI1 directly interacts with FRY2/CPL1 in plant cells. Third, the *shi1* mutation disrupted the interaction between SHI1 and FRY2/CPL1 (Figure 6), which suggests that SHI1 requires FRY2/CPL1 for its function. Forth, *shi1* and *shi4* mutants exhibited very similar expression patterns of stress inducible genes (Figure 3). These evidences strongly support that SHI1-FRY2/CPL1 forms a functional complex to regulate gene expression.

Both SHI1 and FRY2/CPL1 contain domains for interaction with nucleic acids. SHI1 contains five KH domains that have been considered as RNA or single stranded DNA binding domains, while FRY2/CPL1 possesses two dsRNA binding domains. Although both domains were proposed to interact with nucleic acids, our deletion assay for SHI1-FRY2/CPL1 interaction revealed that the third KH domain in SHI1 and the first dsRNA binding domain in FRY2/CPL1 are required for such interaction. This indicates that both KH domain and dsRNA binding domain can mediate protein-protein interaction. Interestingly, we found that, in addition to the third KH domain, the first KH domain in SHI1 is also sufficient to interact with FRY2/CPL1 (Figure 6A). The deletion analysis showing in the Figure 6A also raised a possibility of intramolecular interactions within SHI1 because KH2 inhibits the interactions of KH1 and KH3 with FRY2/CPL1. To test whether intramolecular interactions among the KH domains exist within the SHI1 protein, we carried out a yeast two-hybrid assay for each KH domain. We found that the KH2 can interact with both KH1 and KH3, and KH3 alone can activate the reporter gene expression in yeast cells (Figure S6). Self-activation of the reporter gene expression by the KH3 indicates that the KH3 may be able to interact with and recruit the transcription machinery to the promoter for transcription. These results suggest that SHI1 protein may undergo structural rearrangement upon binding with FRY2/CPL1. This structural rearrangement may promote SHI1 interactions with chromatin and components within the transcription machinery, thus acting as a bridging protein to recruit FRY2/CPL1 to the transcription initiation site through its KH domains that are capable of interacting with nucleic acids and proteins.

SHI1-FRY2/CPL1 complex is involved in mRNA 5′-capping, which is supported by our findings shown in Figure 8. This is consistent with the facts that FRY2/CPL1 functions as a Ser-5 specific CTD phosphatase [10] and the Ser-5 phosphorylation is required for recruiting 5′ capping enzymes [1]. Based on our results, we propose that, at normal growth conditions, the SHI1-FRY2/CPL1 complex is associated with the general transcription machinery and perhaps other unidentified negative regulators at the transcription initiation site of the stress inducible promoter. This repressor complex inhibits the transition from transcription initiation to elongation due to dephosphorylation of the Ser-5 in the CTD and compromising subsequent 5′ capping of the nascent transcripts. According to this model, there might be an abortive transcription at the stress inducible promoter that ensures correct initiation site for transcription and readiness for transcription upon stress treatments. In fact, we detected short transcripts ranging from 40 to about 200 nucleotides that were transcribed from the *AtSOT12* promoter-luciferase fusion gene. Under stress conditions, the repressor complex could readily become an active transcription complex by simply removing the SHI1-FRY2/CPL1 proteins through binding of activators or modifications of the repressor components. The SHI1 transcript level is remarkably reduced by salt stress treatment (Figure 5D), which represents a mechanism of de-repression of a stress inducible gene under stress conditions. Interestingly, amongst the five tested genes, SHI4/FRY2/CPL1 is required for inhibition of capping of all five genes transcripts, while SHI1 displays differential regulation of capping of the five genes. This suggests that SHI4/FRY2/CPL1 does not require SHI1 at all gene loci for modulation of mRNA capping. Another interesting observation is that *shi1* and *shi4* mutations had opposite effects on the stress-induced gene expression of *COR15A* and *COR47* (Figures 3 and S2), while these two mutants showed different but not opposite effects on mRNA capping and polyadenylation site selection of these two genes (Figures 8 and 9). The expression patterns of *COR15A* and *COR47* in *shi1* and *shi4* mutants highly resembles the expression patterns of these two genes in another *shiny* mutant named *shi2* with a mutation in a gene encoding an mRNA splicing factor (Shi lab, unpublished data). Thus, it is possible that reduced induction of *COR15A* by stress treatments in *shi1* and *shi4* mutants is due to mal-splicing of *COR15A* mRNA, whereas increased induction of *COR47* in these two mutants is mainly attributed to elevated mRNA capping. In fact, *shi1* and *shi4* mutants exhibited stronger effects on *COR47* mRNA capping and polyadenylation site selection than *COR15A* (Figures 8 and 9). In spite of the difference in the capping of endogenous genes transcripts, both SHI1 and SHI4 exhibit strong inhibition of capping of the transgene luciferase mRNA (Figures 8). In addition, strong effects of the *shi1* and *shi4* mutations on polyadenylation site selection of the transgene luciferase were also observed (Figure 9). Whether the SHI1-SHI4/FRY2/CPL1 complex is involved in transgene silencing through modulating co-transcriptional processes such as capping and polyadenylation of the transgenes deserves further investigation.

Increased 5′ capping of the luciferase transgene in the *shi1* and *shi4* mutants could explain why the fold change on luciferase activity is significantly higher than the fold change on luciferase transcript level (Figure 1B and 1D). We deduce that, due to more capped luciferase mRNAs in these two mutants than in the wild type, the translation efficiency of the luciferase mRNA is higher in the *shi1* and *shi4* than in the wile type. Substantial difference on the selection of polyadenylation sites between the mutants and wild type (Figure 9) suggests that lacking a functional SHI1 or SHI4 in the mutants may have caused formation of a complex not only with low repression activity but also lacking components that are needed for polyadenylation site selection. These hypotheses need to be further verified. Identification of the components within the repressor complex involving SHI1-FRY2/CPL1 will greatly help our understanding about gene repression, which deserves further study.

## Materials and Methods

### Plant Materials, Mutant Isolation, and Growth Conditions

The firefly luciferase ORF fragment was released from the *RD29A-LUC* construct by digestion with *Hind* III and *Sma* I and inserted into pCAMBIA1381Z at *Hind* III and *Pmal* I by replacing the original GUS gene to form pCAMBIA1381Z-LUC. The promoter and the entire 5′-UTR of At2g03760 was amplified using *Pfu* polymerase (Stratagene) with a forward primer ccccccggggaaggtttccaccttcacactc and a reverse primer aaaactgcagtgttgagacttgagagatcgatca with restriction sites underlined, then inserted into pCAMBIA1381Z-LUC at *Xma* I and *Pst* I to form a transcriptional fusion of *AtSOT12P-LUC*. *Arabidopsis thaliana Columbia-0* plants expressing *AtSOT12P-LUC* were obtained by *Agrobacterium*-mediated transformation using the floral dip method [30]. A homozygous line (named T3 9-1) showing normal growth and development and induced luciferase expression by salt stress treatment was selected as the parental line (referred to as wild type) for mutagenesis using ethyl methane sulfonate (EMS). Seedlings of the M2 generation from the EMS-mutagenized seeds were screened for mutants with altered luciferase expression with or without NaCl treatment by using a charge-coupled device camera (DU434-BV, Andor Technology, Connecticut) according to the previously published protocol [41]. The putative mutants were transferred to soil and subjected to a re-screening process to eliminate the false positives.

### Cold and ABA Tolerance Assays

To determine the sensitivity of seed germination to ABA, the seeds were sterilized for 15 min in 20% Clorox bleach with 0.05% Triton X-100, washed 3–5 times with sterilized water, suspended in 0.3% low melting point agarose and incubated at 4°C for 2 days. The seeds were then planted onto half MS agar medium with different concentrations of ABA and incubated at room temperature under 16 h/8 h fluorescent light cycle. Germination was scored when cotyledon was emerged. The test of plant sensitivity to cold conditions was carried out by transferring two-week old seedlings growing in soil into a 4°C cold room and incubated under 16 h/8 h light cycle.

### Mapping and Identification of the *SHI1* and *SHI4* Locus

For map-based cloning, mutants were crossed with wild-type *Landsberg erecta* and the F1 plants were selfed to generate the F2 seeds. The F2 seedlings showing higher luciferase expression were selected for SSLP marker-assisted genetic mapping. The mutations were identified by sequencing candidate genes. For genetic complementation of the *shi1* mutation, two T-DNA insertion lines SALK_143161 and SALK_001448 of the *SHI1* were obtained from the Arabidopsis Biological Resource Center (ABRC) [42]. Homozygous T-DNA lines were isolated by using a PCR-based method. The *shi1* mutant was crossed with the T-DNA lines and the F1 seedlings resulted from the genetic crosses were subjected to luciferase imaging. For molecular complementation assay, a genomic DNA fragment containing the *SHI1* open reading frame along with 1910 bp of sequence upstream of the translation start codon and 262 bp of sequence downstream of the translation stop codon was amplified from the BAC clone MINB8 using *Pfu* polymerase (Stratagene). The fragment was then inserted into *Bam*H I and *Not* I sites of the Gateway entry vector pENTR1A and was recombined into the plant transformation Gateway vector pMDC100 (ABRC stock number CD3-746) with Gateway LR Clonase II Enzyme Mix (Invitrogen) following the manufacturer's instruction. The resulting construct was transformed into *shi1* mutant and the T2 transgenic lines were used for luciferase imaging.

### Promoter-GUS and Subcellular Localization Analysis

The promoter region of the *SHI1* gene with 1847 bp upstream of the translation start codon was amplified using from the BAC clone MINB8 using *Pfu* polymerase (Stratagene) and inserted into *Bam*H I amd *Pst* I sites of the binary vector pCAMBIA1381Z to create a transcriptional fusion of the *SHI1* promoter with the GUS reporter gene. The resulting construct was transferred into Colunbia-0 wild-type plants by the floral dip method [30]. The T2 seedlings were stained with X-Gluc staining buffer (10 mM Tris pH 7.0, 10 mM EDTA, 0.1% Triton X-100 and 2 mM 5-bromo-4-chloro-3-indolyl-beta-D-glucuronic acid) for 12–24 hours at 25°C, followed by incubating in 70% ethanol to remove chlorophyll. For protein subcellular localization assay, the ORF of the *SHI1* gene was recombined from pENTR1A into the PMDC43 vector [43] to create SHI1-GFP fusion and the ORF of FRY2 was recombined into pEarleyGate103 [44] for FYR2-GFP fusion. The fusion constructs were transformed into Arabidopsis by the floral dip method [30] and the T2 transgenic plants were used to examine the location of the GFP fluorescence using confocal microscope.

### Gene Expression Assay

For gene expression study, wild-type and *shi1* mutant seeds were planted on half MS agar medium. Ten-day-old seedlings were subjected to NaCl, ABA and cold treatments as previously described [11]. To study the expression of *SHI1*, 10-day-old wild type seedlings were treated with different stress conditions following the method described by Chung et al. [45] and different parts of plants were collected from soil-growing plants. Total RNA was extracted from the seedlings and analyzed by RNA blotting. The DNA probes for the tested genes were PCR-amplified by using the following primers: *SHI1*, atggagagatctagatccaagagaaactac and tactgctgtcttgttgtccctgag; *COR15A*, aaagaaagcttcagatttcgtg and agaatgtgacggtgactgtgg; *KIN1*, tctcttctcatcatcactaacc and tttggggagtttgatctttcgc; *COR47*, cgacgagaaagcagaggattc and cgaggtgatcatgtgaataacg; *CBF1*, cgatagtcgtttccatttttgt and ttgctagattcgagacgagcc; *CBF2*, ttcgatttttatttccatttttgg and ccaaacgtccttgagtcttgat; *CBF3*, taaaactcagattattatttccattt and aggagccacgtagagggcc; *DREB2A*, caaaacaatatgaagctttttgg and agtgtgtattattcattcctg; *LUC*, tggagagcaactgcataagg and tgacgcaggcagttctatgc; *AtSOT12*, atgtcatcatcatcatcagttcctg and tcaagaagaaaatttaagaccagaacc; and *β-tubulin* gene (*AT5G23860.1*), cgtggatcacagcaatacagagcc and cctcctgcacttccacttcgtcttc. DNA probes were radioactively labeled with [α-^32^P] dCTP using the Prime-It II Random Primer Labeling Kit (Stratagene).

Quantitative RT-PCR to determine the expression levels of *LUC*, *CBF3*, *COR15A* and *COR47* was carried out as follows. 2 µg of total RNA isolated with Plant RNA Purification Reagent (Invitrogen) was reverse transcribed into cDNAs using the avian myeloblastosis virus (AMV) reverse transcriptase (Promega) and oligo dT_(15)_. The cDNAs were used as template for quantitative real-time PCR using ABI PRISM 7500 Real-Time PCR Systems (Applied Biosystems) and the iTaq SYBR Green Supermix with ROX kit (Bio-Rad, Hercules, CA, USA). The following primers were used: tggagagcaactgcataagg and gttcacctcgatatgtgcatctgt for *LUC*; cgacgacggatcatggcttc and ctccataacgatacgtcgtcatc for *CBF3*; agatggtgagaaagcgaaagactac and gaactctgccgccttgtttg for *COR15A*; ttcaccagctgtcacgtcca and cttctcctccggatgttcca for *COR47*; ccgagtatgatgaggcaggtc and cccattcataaaaccccagc for actin2 for RNA normalization.

### Yeast Two-hybrid Assay

The yeast two-hybrid screening with *Saccharomyces cerevisiae* strainY190 was performed in accordance with the previously described protocol [46]. The cDNA library for yeast two-hybrid screening was obtained from the ABRC (stock no. CD4-300) [47]. The ORF of *SHI1* from the cDNA clone G16563 (ABRC) was recombined into the destination vectors pDEST-GADT7 (prey vector) and pDEST-GBKT7 (bait vector) [48] using LR clonase kit (Invitrogen). Self-activation test verified that the bait pGBKT7–SHI1 plasmid has no autonomous activity of the reporter genes in the yeast strain Y190. For screening, yeast cells harboring the bait construct were transformed with the cDNA library and plated onto synthetic high-stringency selection medium lacking tryptophan, leucine and histidine supplemented with 25 mM 3-amino-1, 2, 4-triazole (Sigma). The putative positive cDNA clones were further confirmed by the β-galactosidase assay and tested for specificity by co-transformation into Y190, either alone or in combination with the empty pDEST-GBKT7 vector. The cDNA inserts from positive clones were sequenced using a Big-Dye Terminator Cycle Sequencing Kit (Applied Biosystems) and the ABI 3100 DNA sequencer.

Deletion analysis was used to determine the domains required for interaction between SHI1 and FRY2 in the yeast strain Y190. For a series deletions of the carboxyl terminus of SHI1, the forward primer is acgcgtcgacatggagagatctagatccaagagaaactac with *Sal* I restriction site (underlined), the reverse primers are cggaattcgtaacaagtggtaccgccattctg, cggaattcgttgttattaaattatctttatccggaataag, cggaattctcggagtagacattctcaccaactg, cggaattccctccaccaggtctaactccac with *Eco*R I restriction site (underlined); for the amino terminal deletions of SHI1, the reverse primer is gatatctcggtccatcctcttgtatgctcaaaatgaag with *Eco*R V restriction sites (underlined), the forward primers are acgcgtcgacatgagtgttcatgacaggattttggaga, acgcgtcgacatgtcatctcgtctaagggagagtcagc, acgcgtcgacatgttgcacattcaaactcagatcatagat and acgcgtcgacatggagatcagagctgctcggga with *Sal* I restriction sites (underlined). For the Carboxyl terminal deletions of FRY2, the forward primer is acgcgtcgacatgcttcatgagaatcgcaggc with *Sal* I restriction sites (underlined), the reverse primers are cggaattcccagtgtgcctcatagaaccttct, cggaattcgctaaattctgtatagaagcttcagcag and cggaattcgtctccgttgctgagacacttcg with *Eco*R I restriction sites (underlined); for the amino terminal deletions of FRY2, the reverse primer is cggaattcgcctccttcagtcttctctccac with *Eco*R I restriction sites (underlined), the forward primers are acgcgtcgac atgacttcagctgatgttctacacgga, acgcgtcgacatggctgatggatatatgcgtgcaa, acgcgtcgac atgggctccattactgcactcaggg and acgcgtcgacatgtccagtgtgagatcaatgcttgg with *Sal* I restriction sites (underlined). The entire ORF of FRY2 was amplified with the primers acgcgtcgacatgtatagtaataatagagtagaagtgtttcatggt with *Sal* I restriction site (underlined) and ataagaatgcggccgcgagtatcttcccgaagatggca with *Not* I restriction site (underlined) without stop codon from cDNA using *Pfu* polymerase (Stratagene). The PCR fragments were cloned into the Gateway entry vector pENTR1A (Invitrogen), then recombined into the destination vectors pDEST-GADT7 and pDEST-GBKT7, respectively, using LR clonase kit (Invitrogen). The resulting plasmids were transformed into Y190 containing pGBKT7-FRY2 or pGBKT7-SHI1 to test interactions between different forms of SHI and FRY2.

### Protein Pull-down Assay

The ORF of *SHI1* without start codon was amplified with the primers cgggatccgagagatctagatccaagagaaactaccac with restriction site *Bam*H I (underlined) and cggaattccggtccatcctcttgtatgctc with restriction site *Eco*R I (underlined) using *Pfu* polymerase (Stratagene) and was cloned into pGEX2T to create a GST-SHI1 fusion construct. The resulting plasmid was transformed into *E. coli* Rosetta-gami 2 host strains (Novagen) and individual colonies were inoculated in LB medium and grown at 37°C overnight. The cultures were then diluted to approximately OD600 of 0.1 and grown at 37°C until they reached OD600 0.5–1.2. The protein expression was induced with 0.5 mM isopropyl β-D-1-thiogalactopyranoside for an additional 16–24 h at 30°C on a shaker. Bacterial cells were pelleted and then resuspended in the lysis buffer (50 mM Tris–HCl pH 8.0; 100 mM NaCl and 1 mM EDTA). After sonication and centrifugation, the recombinant protein in the supernatant was purified by using GST Bind Resin (Novagen).

The fragment of *FRY2* encoding the first dsRNA binding domain (666–855 aa) in the pENTR1A vector was recombined into the pDEST17 vector to generate a HIS-tagged protein under the control of the T7 promoter. The plasmid was used for *in vitro* transcription-coupled translation in the presence of [^35^S]-Methionine using the TNT Quick Coupled Transcription/Translation Systems (Promega) according to the manufacturer's instructions. The purified GST-SHI1 and *in vitro* translated peptides of FRY2 were analyzed by protein gel blotting using anti-GST and anti-HIS antibodies, respectively. Pull-down experiments were performed as follows. GST (as negative control) or GST-SHI1 protein was immobilized to GST Bind Resin (Novagen), which was incubated with [^35^S]-labeled peptides of FRY2 (HIS-cFRY2) for 30 min at room temperature in 100 µL of the binding buffer (20 mM Tris-HCl, pH 7.2, 10 mM MgCl_2_, and 2 mM DTT). After extensive washing with PBS buffer, the beads were resuspended in 50 µL of the protein loading buffer and the proteins were resolved in 12% SDS–polyacrylamide gel. The gels were dried and placed in direct contact with the X-ray film (Kodak) in dark at room temperature for autoradiography.

### Interaction of SHI1 and FRY2 in Plants

For split luciferase (*Renilla reniformis*) complementation assay, the *SHI1* ORF was amplified with a forward primer acgcgtcgacatggagagatctagatccaagagaaactac with *Sal* I restriction site (underlined) and a reverse primer gatatctcggtccatcctcttgtatgctcaaaatgaag with eliminated stop codon using *Pfu* polymerase (Stratagene). The PCR fragment was digested with *Sal* I only and inserted into pENTR1A after digested with *Sal* I and *Eco*R V. The *SHI1* ORF was then recombined into pDUExAn6 vector and the *FRY2* ORF in the pENTR1A vector was recombined into pDUEXDc6 vector [31]. For split YFP complementation assay, the *SHI1* ORF was amplified with a forward primer ccgctcgagatggagagatctagatccaagagaaactac with a *Xho* I restriction site (underlined) and a reverse primer cggaattccggtccatcctcttgtatgctc with a *Eco*R I restriction site (underlined) without stop codon using *Pfu* polymerase (Stratagene) and inserted into pSAT4A-nEYFP-N1 [49]. The *FRY2* ORF was fused to the C-terminal of YFP in the pSAT4A-cEYFP-N1 [49], which was amplified using a forward primer ccgctcgagatgtatagtaataatagagtagaagtgtttcatggt with a *Xho* I restriction site (underlined) and a reverse primer cggaattcagagtatcttcccgaagatggca with a *Eco*R I restriction site (underlined) without stop codon using *Pfu* polymerase (Stratagene). Preparation of Arabidopsis protoplasts and transformation of the constructs into the protoplasts with PEG-mediated method were performed essentially following Yoo et al. [50]. The protoplasts transformed with the constructs for split luciferase assays were transferred to a 96-well plate and subjected to luciferase imaging by using the luciferase imaging system (DU434-BV, Andor Technology, Connecticut). Fluorescence imaging of YFP in the split YFP assay and Hoechst 33342 staining was carried out by using an inverted fluorescence microscope.

For co-immunoprecipitation (Co-IP) experiments, the ORF of *SHI1* was recombined into the pEarleyGate vector 203 [44] for Flag-SHI1 fusion. The ORF of *FRY2* was recombined into pEarleyGate 205 [44] for FYR2-cTAP fusion. The fusion constructs were transformed into Arabidopsis by floral dip method [30]. The T2 transgenic plants of Flag-SHI1 crossed with FYR2-cTAP plants and the F1 seedlings were used for protein isolation. Co-IP assay was carried out essentially following the previously described method [44].

### Detection of mRNA Capping

RLM-qRT-PCR was used to analyze the relative ratio of capped transcripts in total transcripts of *LUC*, *AtSOT12*, *At5G25280*, *COR15A* and *COR47* genes. This method was developed based on the RLM-RACE method. Total RNA was first treated by using Firstchoice RLM-RACE Kit (Invitrogen) including cipping, decapping, RNA adapter ligation, and cDNA synthesis through reverse transcription using random primers, as described in the manufacturer's manual. The cDNAs were then used as templates for Quantitative real-time PCR analysis of the capped mRNA and total mRNA of an individual gene. Briefly, 10 µg of total RNA isolated with Plant RNA Purification Reagent (Invitrogen) was treated by Calf Intestine Alkaline Phosphatase (CIP) to remove free 5′-phospates from uncapped mRNA, rRNA or tRNA and leave full-length, capped mRNA intact. This step prevents the uncapped mRNA from ligation with the RNA adapter described below. The CIP-treated RNA was treated by Tobacco Acid Pyroposphatase (TAP) to remove the cap structure from the full-length, capped mRNA and leave a monophosphate at the 5′-end of them. An adapter oligonucleotide (gcugauggcgaugaaugaacacugcguuugcuggcuuugaugaaa) was then ligated to full-length, decapped mRNA using T4 ligase. Finally, 1 µg of treated RNA was reverse transcribed using the M-MLV reverse transcriptase and Random Decamers. The constructed cDNA was diluted 10 times, 20 times and 40 times and used as templates for Quantitative real-time PCR. Quantitative real-time PCR was performed by using ABI PRISM 7500 Real-Time PCR Systems (Applied Biosystems) and the iTaq SYBR Green Supermix with ROX kit (Bio-Rad, Hercules, CA, USA). The following primers were used: tgatggcgatgaatgaacactg and tagaggatagaatggcgccg for capped *LUC* mRNA, gctggagagcaactgcataagg and tagcttctgccaaccgaacg for total *LUC* mRNA, tgatggcgatgaatgaacactg and gcaggaactgatgatgatgatgac for capped *AtSOT12* mRNA, cttgggagatgaagatctgacaca and cgtttttggcagatcaagattc for total *AtSOT12* mRNA, gctggagagcaactgcataagg and gacggtgatttggatcggag for capped *At5G25280* mRNA, atcaaacggatctgcttcgc and acctgacgacgacggagatg for total *At5G25280* mRNA, tgatggcgatgaatgaacactg and agccataccagtgagaacagctc for capped *COR15A* mRNA, gagctgttctcactggtatggct and ttctggccgactctgacagc for total *COR15A* mRNA, tgatggcgatgaatgaacactg and gaccgttggtgtctcgtgct for capped *COR47* mRNA, aagaacaacgttcccgagca and cgttgtctcttgaggtttcacttc for total *COR47* mRNA, caaccaatcgtgtgtgacaatg and acagccctgggagcatcat for actin2 for RNA normalization. Note that the forward primer used for capped mRNA amplification was derived from the RNA adapter, so the PCR only specifically amplified the decapped and RNA adapter ligated mRNA of an individual gene. The ratio between the capped transcripts and total transcripts was calculated and presented as the relative capping ratio of each gene shown in Figure 8A.

5′ RACE was used to analyze the capping ratio of the luciferase mRNAs. Briefly, 2 µg of total RNA isolated with RNeasy Plant Mini Kit (Qiagen) was reverse transcribed using the avian myeloblastosis virus (AMV) reverse transcriptase (Promega) following the manufacture's instruction. The first strand cDNA purified with the PCR purification kit (Qiagen) was added with a Poly (dA) tail using terminal transferase (New England Biolabs). The cDNA having a poly (A) tail was amplified with a primer ctgatctagaggtaccggatcc-dT_(17)_ and the luciferase gene specific primer gtttcatagcttctgccaacc for 5′RACE using GoTaq DNA Polymerase (Promega). The PCR products were further amplified with an adaptor primer ctgatctagaggtaccggatcc and the nested luciferase gene specific primer gcagttgctctccagcggtt. The PCR fragments were then cloned into pGEM-T Easy vector (Promega) and sequenced using a Big-Dye Terminator Cycle Sequencing Kit (Applied Biosystems) on the ABI 3100 DNA Sequencer following a standard protocol. At least 20 independent clones were sequenced and the additional G at the very end of each cDNA was counted as from a capped mRNA.

### Determination of Polyadenylation Sites

The polyadenylation sites of the mRNAs were determined by using 3′ RACE method. Briefly, 2 µg of total RNA isolated with Plant RNA Purification Reagent (Invitrogen) was reverse transcribed using the avian myeloblastosis virus (AMV) reverse transcriptase (Promega) and oligo dT_(15)_. The cDNA corresponding to the 3′ end of the target gene mRNA was then synthesized by PCR amplification using the adaptor-dT_(15)_ (ctgatctagaggtaccggatcc-dT_(15)_) and the target gene specific primer (*LUC*: gttttggagcacggaaagacg; *AtSOT12*: ttgccaaatggaatagagactaaaac; *At5G25280*: gcttatgagcctcgtcgtagtaga; *COR15A*: caaacaaggcggcagagttc; *COR47*: ttcaccagctgtcacgtcca). The PCR product was then used as template for a nested PCR amplification by using the adaptor primer ctgatctagaggtaccggatcc and the nested target gene specific primer (*LUC*: cgtggattacgtcgccagtc; *AtSOT12*: ggagagatactttgagtgagtcattgg; *At5G25280*: tagactcgcatctatgtcgaaagc; *COR15A*: gttcgcggagggtaaagcag; *COR47*: tggaacatccggaggagaaga). The PCR-amplified fragments were then cloned into pGEM-T Easy vector and sequenced using a Big-Dye Terminator Cycle Sequencing Kit (Applied Biosystems) on the ABI 3100 DNA Sequencer following a standard protocol. At least 20 independent clones were sequenced and the polyadenylation sites were scored according to the sites linking with the PolyA.

## Supporting Information

Figure S1 Detection of salt-induced expression of the luciferase transgene driven by the *AtSOT12* promoter. (A) Luciferase activity measurements of the homozygous line expressing *AtSOT12P-LUC* transgene. Values are means ± SD (n = 3). (B) Northern blot showing induction of the luciferase transgene and *AtSOT12* gene by NaCl treatment. Tubulin is shown as a loading control. Ctrl. Control without salt treatment; NaCl, 200 mM NaCl for 5 h.
(TIF)

Figure S2 Quantitative RT-PCR showing the transcript levels of stress-inducible genes in wild type (WT), *shi1* and *shi4* mutants in response to different abiotic stress treatments. Cold, 0°C for 12 h; NaCl, 200 mM NaCl for 12 hr; ABA, 100 µM ABA for 3 h. Values are mean ± SD (n = 3).
(TIF)

Figure S3 Split-YFP assay for SHI1 and FRY2 interaction. Left panel, YFP fluorescence in Arabidopsis protoplasts transformed with the constructs of SHI1-nYFP and FRY2-cYFP; Right panel, Nucleus staining by Hoechst 33342. Note that protoplasts were also transformed with vectors only, SHI1-nYFP+cYFP vector and nYFP vector+SHI1-cYFP as controls and no YFP was detected in these control experiments.
(TIF)

Figure S4 Northern blotting showing the transcription levels of *At5g25280* gene in wild type (WT), *shi1* and *shi4* mutants. Tubulin is shown as a loading control.
(TIF)

Figure S5 Characterization of the cap-binding protein eIF4E (At4g18040). (A) Binding assay of GST-eIF4E protein with capped (left panel) and uncapped (right panel) mRNAs synthesized by *in vitro* transcription. The binding assay was essentially followed Choi and Hagedorn [33]. Capped and uncapped mRNAs were synthesized by *in vitro* transcription and the *in vitro* synthesized and radiolabeled mRNAs were mix with GST-eIF4E agarose beads and loaded into a column, washed with 1 mL 1X binding buffer twice followed by washing with 1 mL 500 µM GDP three times. mRNAs were eluted with 1 mL binding buffer containing 1 mM m^7^GDP. The beads were extracted with equal volumes of phenol/chloroform. mRNA in each sample was precipitated by ethanol. RNA was analyzed by gel electrophoresis with 6% polyacrymide containing 7 M urea followed by autoradiography. Lane 1 shows unbound mRNAs most likely without cap; lanes 2–6 show non-specific binding of eIF4E with mRNAs washed out with biding buffer and 500 µM GDP; lanes 7–8 show specific binding of eIF4E with capped mRNAs. No binding activity was detected with uncapped mRNAs (lanes 7–8 on the right panel). (B) Binding kinetics of eIF4E with capped mRNA. The calculated binding constant is 0.18 nM.
(TIF)

Figure S6 Yeast two-hybrid assays showing interactions amongst the KH domains in SHI1 protein. + means interaction and − means no interaction. Note that the KH3 has self activation activity when fused with the BD.
(TIF)
