# Supplementary figures and images for: The Arabidopsis RNA Binding Protein with K Homology Motifs, SHINY1, Interacts with the C-terminal Domain Phosphatase-like 1 (CPL1) to Repress Stress-Inducible Gene Expression
Source: PLoS Genet. 2013 Jul 11;9(7):e1003625. doi: 10.1371/journal.pgen.1003625 (PMC3708844; doi:10.1371/journal.pgen.1003625)

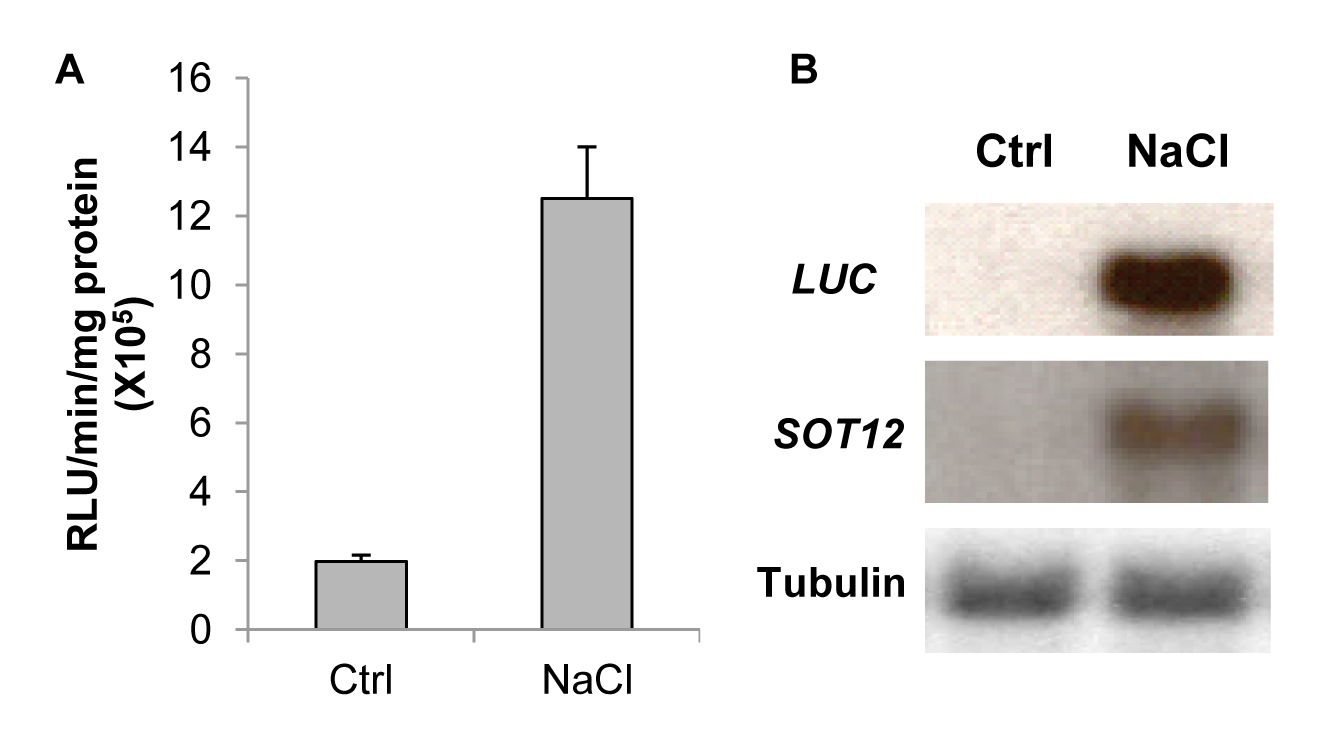

Supplement: Figure S1 — Detection of salt-induced expression of the luciferase transgene driven by the AtSOT12 promoter. (A) Luciferase activity measurements of the homozygous line expressing AtSOT12P-LUC transgene. Values are means ± SD (n = 3). (B) Northern blot showing induction of the luciferase transgene and AtSOT12 gene by NaCl treatment. Tubulin is shown as a loading control. Ctrl. Control without salt treatment; NaCl, 200 mM NaCl for 5 h. (TIF) [file pgen.1003625.s001.tif]

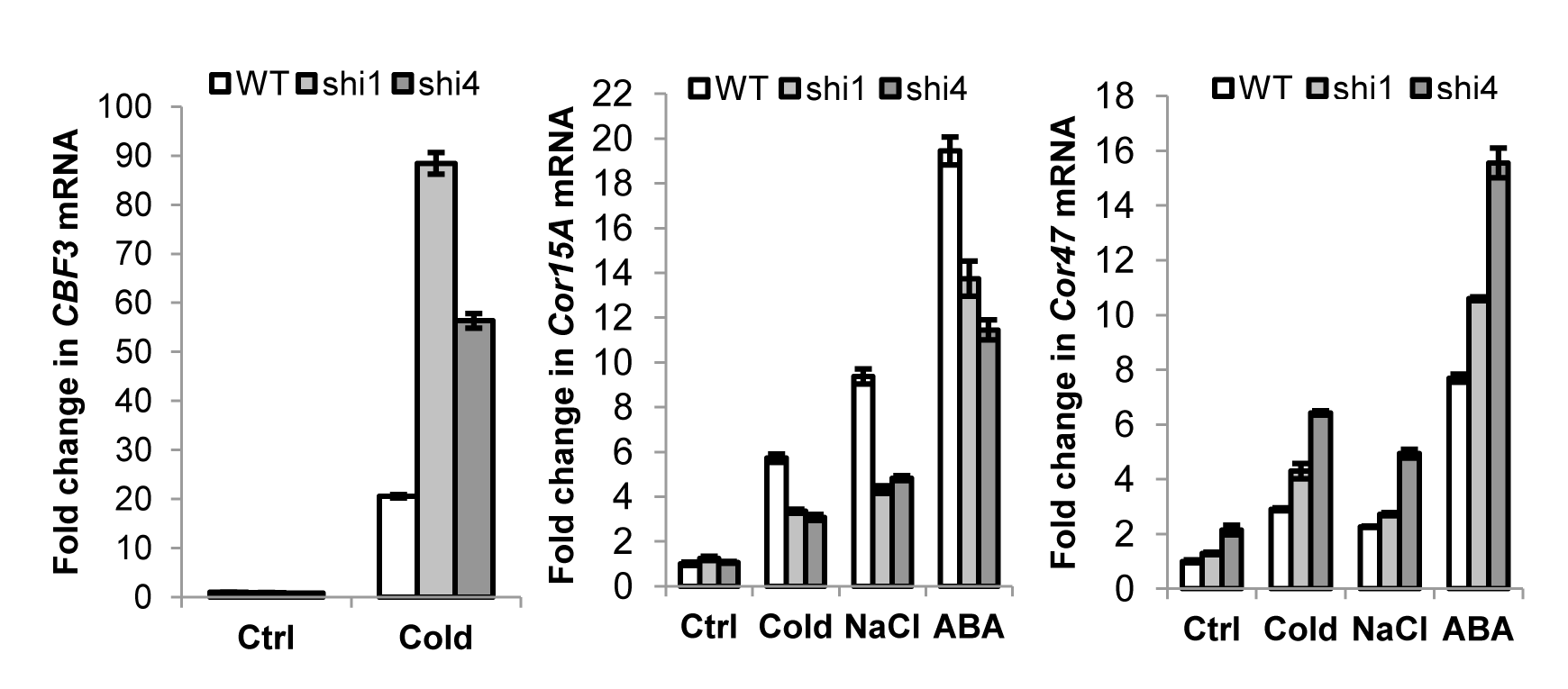

Supplement: Figure S2 — Quantitative RT-PCR showing the transcript levels of stress-inducible genes in wild type (WT), shi1 and shi4 mutants in response to different abiotic stress treatments. Cold, 0°C for 12 h; NaCl, 200 mM NaCl for 12 hr; ABA, 100 µM ABA for 3 h. Values are mean ± SD (n = 3). (TIF) [file pgen.1003625.s002.tif]

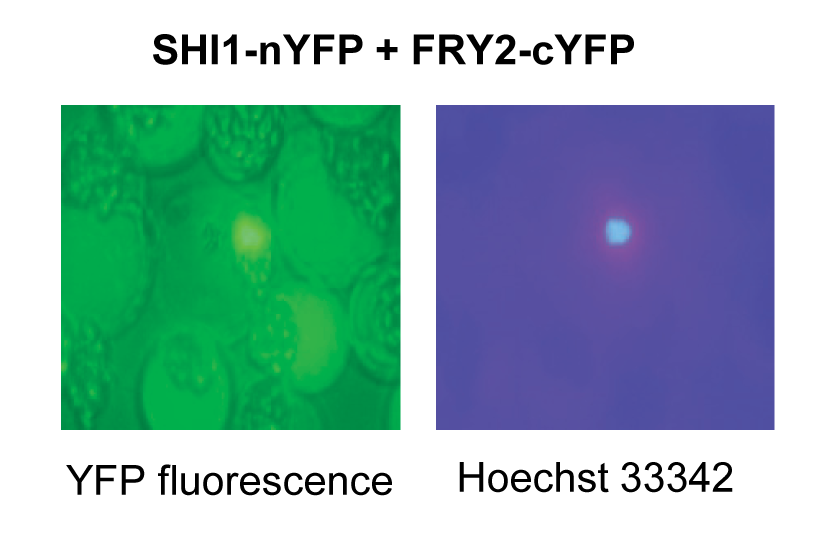

Supplement: Figure S3 — Split-YFP assay for SHI1 and FRY2 interaction. Left panel, YFP fluorescence in Arabidopsis protoplasts transformed with the constructs of SHI1-nYFP and FRY2-cYFP; Right panel, Nucleus staining by Hoechst 33342. Note that protoplasts were also transformed with vectors only, SHI1-nYFP+cYFP vector and nYFP vector+SHI1-cYFP as controls and no YFP was detected in these control experiments. (TIF) [file pgen.1003625.s003.tif]

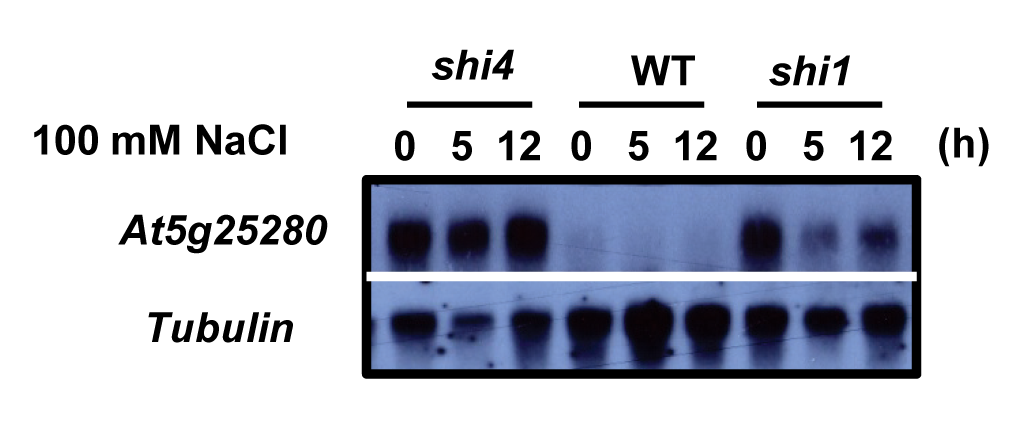

Supplement: Figure S4 — Northern blotting showing the transcription levels of At5g25280 gene in wild type (WT), shi1 and shi4 mutants. Tubulin is shown as a loading control. (TIF) [file pgen.1003625.s004.tif]

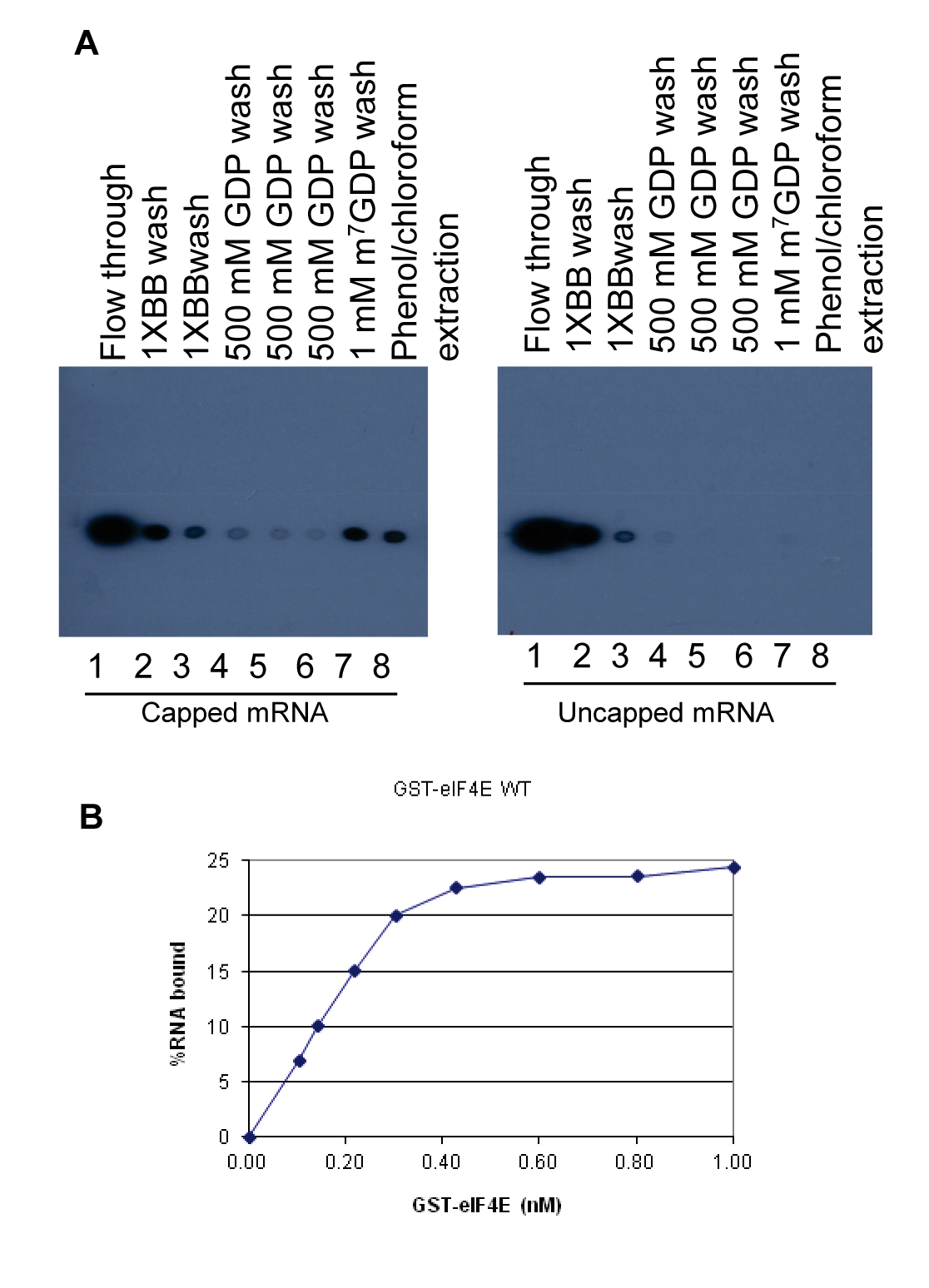

Supplement: Figure S5 — Characterization of the cap-binding protein eIF4E (At4g18040). (A) Binding assay of GST-eIF4E protein with capped (left panel) and uncapped (right panel) mRNAs synthesized by in vitro transcription. The binding assay was essentially followed Choi and Hagedorn [33]. Capped and uncapped mRNAs were synthesized by in vitro transcription and the in vitro synthesized and radiolabeled mRNAs were mix with GST-eIF4E agarose beads and loaded into a column, washed with 1 mL 1X binding buffer twice followed by washing with 1 mL 500 µM GDP three times. mRNAs were eluted with 1 mL binding buffer containing 1 mM m7GDP. The beads were extracted with equal volumes of phenol/chloroform. mRNA in each sample was precipitated by ethanol. RNA was analyzed by gel electrophoresis with 6% polyacrymide containing 7 M urea followed by autoradiography. Lane 1 shows unbound mRNAs most likely without cap; lanes 2–6 show non-specific binding of eIF4E with mRNAs washed out with biding buffer and 500 µM GDP; lanes 7–8 show specific binding of eIF4E with capped mRNAs. No binding activity was detected with uncapped mRNAs (lanes 7–8 on the right panel). (B) Binding kinetics of eIF4E with capped mRNA. The calculated binding constant is 0.18 nM. (TIF) [file pgen.1003625.s005.tif]

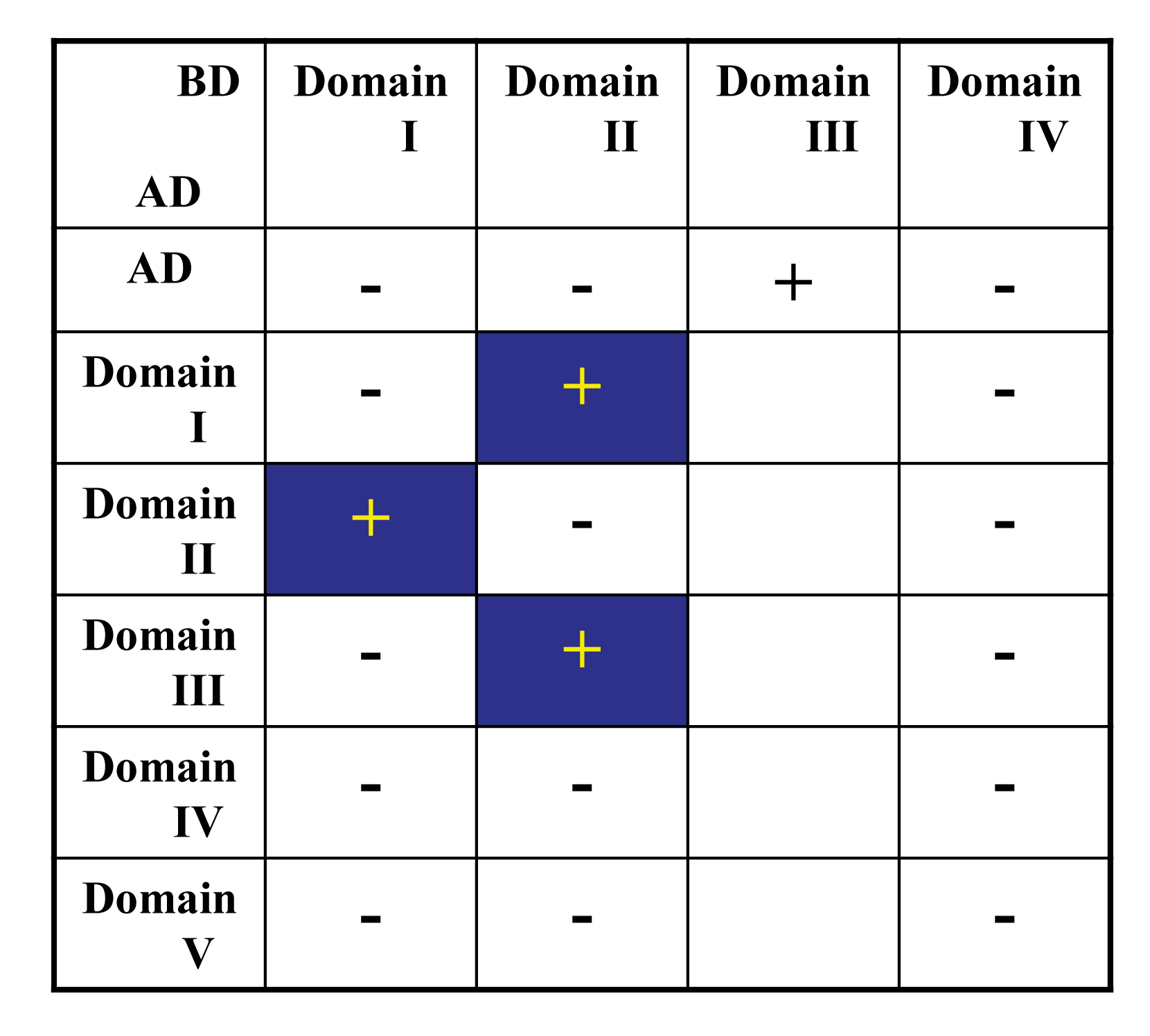

Supplement: Figure S6 — Yeast two-hybrid assays showing interactions amongst the KH domains in SHI1 protein. + means interaction and − means no interaction. Note that the KH3 has self activation activity when fused with the BD. (TIF) [file pgen.1003625.s006.tif]
